# Two domesticated species of rice shaped the population structure of *Xanthomonas oryzae* pv. *oryzae* in Africa

**DOI:** 10.1371/journal.ppat.1014015

**Published:** 2026-09-17

**Authors:** Ian Lorenzo Quibod, Coline Sciallano, Florence Auguy, Laurent Brottier, Alexis Dereeper, Diariatou Diagne, Amadou Diallo, Hinda Doucouré, Solely Issaka Mayaki, Ibrahim Keita, Lazeni Konate, Hamidou Tall, Cheick Tékété, Sylvain Zougrana, Mathilde Hutin, Ousmane Koita, Daoudou Kone, Soungalo Sarra, Valérie Verdier, Issa Wonni, Boris Szurek, Sébastien Cunnac, Alvaro L. Pérez-Quintero

**Affiliations:** 1 Plant Health Institute of Montpellier, University of Montpellier, CIRAD, INRAE, Institut Agro, IRD, Montpellier, France; 2 Laboratoire de Biologie Moléculaire Appliquée, Faculté des Sciences et Techniques, Université des Sciences et Techniques et des technologies de Bamako, Bamako, Mali; 3 Institut Supérieur Agronomique et Vétérinaire « Valéry Giscard d’Estaing » de Faranah (ISAV « VGE »/F), Faranah, République de Guinée; 4 Pathogens Genomic Diversity Network Africa, Bamako, Mali; 5 Université de Tilaberi, Tillabéri, Niger; 6 Laboratoire de Biologie Moléculaire Appliquée, Faculté de Médecine et d'Odontostomatologie, Université des Sciences et Techniques et des Technologies de Bamako, Bamako, Mali; 7 Institut Sénégalais de Recherches Agricoles, Tambacounda, Senegal; 8 Centre National de Recherche Scientifique et Technologique (CNRST)/ Institut de l’Environnement et de Recherches Agricoles (INERA), Bobo Dioulasso, Burkina Faso; 9 Laboratoire de Biologie Moléculaire Appliquée et Centre de Recherche en Santé Globale, Bamako, Mali; 10 Laboratoire de Physiologie Végétale, Université Félix Houphouët-Boigny, Abidjan, Côte d'Ivoire; 11 Centre Régional de Recherche Agricole de Niono, Institut d'Economie Rurale, Bamako, Mali; The Ohio State University, UNITED STATES OF AMERICA

## Abstract

African rice (*Oryza glaberrima*) was independently domesticated in West Africa around 3000 years ago, and has long been intertwined in the history of the region. Asian rice (*Oryza sativa*), which was introduced in Africa when European settlers arrived, gradually replaced African rice and has since dominated rice cultivation in the continent. Domesticated rice species are affected by bacterial leaf blight (BLB), which is caused by the pathogen *Xanthomonas oryzae* pv. *oryzae* (*Xoo*). Here we show that the bacterial leaf blight pathogen in Africa (AfXoo) belongs to a distinct phylogroup from the one circulating in Asia (AsXoo), and has a different evolutionary history. Analysis of 87 AfXoo genomes identified five main populations, including highly clonal ones, and a more diverse and recombinant population. Tip-dating analysis revealed that the AfXoo population went through a period of expansion, then decline and more recent recovery. We hypothesize this followed the rise and fall of African rice, and that the introduction of *O. sativa* served as a bottleneck leading to the emergence of current AfXoo populations. We show that AfXoo has a highly conserved repertoire of type III effectors (T3E), but that nonetheless there is variation especially between populations. In the case of transcription activator-like effectors (TALEs), variation can arise quickly through rearrangements, and we hypothesize that the TALE repertoire of AfXoo has been selected to allow the bacteria to colonize both species of cultivated rice found in the continent. Our research provides an attempt to decipher the genetic history of bacterial blight in West Africa, and its past and present impact on rice cultivation in the region.

## Author summary

For thousands of years, rice cultivation has been an integral part of African agriculture. However, the cultivation of the locally domesticated African rice cultivar (*Oryza glaberrima*) has gradually shifted towards Asian rice varieties (*Oryza sativa*), likely altering associated microbe populations. One of these microbes is the causal agent of bacterial leaf blight, *Xanthomonas oryzae* pv. *oryzae* (*Xoo*). We performed a population genomics analysis to understand the evolutionary history of African strains of *Xoo* (AfXoo). We show that current populations of AfXoo have relatively low genetic diversity, have distinct virulence genes, and have historically gone through expansion and decline. We propose AfXoo was first adapted to African rice at least a thousand years ago. The introduction of *O. sativa* then shaped the recent population dynamics of AfXoo. Our results highlight the interplay between crop domestication and cultivation and pathogen evolution.

## Introduction

Rice has become one of the staple crops in the majority of African countries due to increased demand and consumption, with about 40% of agricultural areas dedicated to rice cultivation [1]. Rice cultivation in Africa can be divided into two periods. The first corresponds to a time during which African rice cultivation was isolated from the rest of the world and centered on the use of *Oryza glaberrima* Steud, a variety that resulted from the domestication of the wild rice *Oryza barthii* in West Africa about 3000 years ago [2]. Its domestication has been proposed to have either a single origin in the inner Niger River Delta [3] or in multiple locations in West and Central Africa [4]. The second period is defined by the introduction of Asian rice (*Oryza sativa*) in West Africa around the 16th century by the Portuguese [5]. The establishment of *O*. *sativa* has significantly reduced the once thriving indigenous African rice industry throughout West Africa since its introduction [5].

Cultivated rice is under constant threat from pests and diseases, which accounts for at least 30% of reduction in yield [6]. One of the major contributors to the loss of harvested rice is Bacterial Leaf Blight (BLB), caused by the gram negative bacterium *Xanthomonas oryzae* pv. *oryzae* (*Xoo*). The disease was first documented in Japan in 1884, and has since decimated rice-growing areas in tropical and subtropical Asian and African countries [7]. In Africa, in particular, BLB was first monitored in the late 70s in Mali [8], and has since been considered endemic in the region [9]. Strains of *Xoo* gathered in Africa (AfXoo) are genetically distinct from their well studied Asian (AsXoo) counterparts [10,11]. Thus, it is important to differentiate the BLB pathogens, especially since there are currently two ongoing epidemics of AsXoo introduced in East African countries including Kenya, Tanzania, Uganda and Madagascar [12–14]. In addition to AfXoo and AsXoo, the *Xanthomonas oryzae* species includes a southern cutgrass (*Leersia hexandra*) pathogen (pathovar (pv.) *leersiae*, *Xol*), a non-vascular rice-infecting pathovar (pv. *oryzicola*, *Xoc*), and non-pathogenic unclassified strains [15,16].

*Xoo*, a vascular pathogen, enters rice through hydathodes or wounds and colonizes the xylem tissues [17]. The pathogen efficiently hijacks host cell processes through secretion of type III effector (T3E) proteins to bypass host recognition and divert nutrients [18]. *Xoo* T3Es can be classified in two groups: Xanthomonas outer proteins (Xop) involved in suppressing immunity [19], and Transcription Activator-Like Effectors (TALEs) involved in inducing physiological changes in the host that increase susceptibility [20]. TALEs are able to bind to the host genome at positions predefined by the variability of certain residues in TALE-specific amino acid repeat sequences (variable di-residues, RVDs). Once bound to specific sequences, called Effector Binding Elements (EBEs), TALEs will induce the expression of neighboring genes, for example SWEET genes (sugar transporters), which when expressed can benefit pathogen colonization [21]. Plants have evolved defense mechanisms against these effectors. For instance, TALEs can be ‘trapped’ by resistance (R) genes promoters [22–24], or recognized by NLRs [25,26]. Also, changes in the promoter sequence of the target gene impair the binding of corresponding TALE, preventing susceptibility gene induction thus protecting the plant against BLB [27,28]. TALEs display high diversity in sequence and function, linked to their propensity for recombination, insertions and deletions in the repeat region [29]. Depending on the phylogroup, TALE repertoire (TALome) varies, with AfXoo carrying less TALEs (around 8–9 per strain) as compared to AsXoo (between 13–20 per strain) [16]. Notably, despite significant differences in TALome and RVD sequences, AfXoo and AsXoo have converged into targeting some susceptibility genes (*SWEET14*, *OsTFX1*) while others are specific to either phylogroup [15].

Having insight into a pathogen’s population diversity and evolutionary potential, and means of spread is crucial for their management through the use of appropriate varieties and the forecasting of epidemics [30]. Evolutionary dynamics of the *Xoo* population have been relatively well explored for the Asian phylogroup through genomics [31–34]. For example, the sequencing of isolates collected in the Philippines revealed that adaptation to BLB-resistant rice cultivars carrying the *Xa4* allele has led to diversification of the *Xoo* populations in the region [32]. Other studies showed that recombination played a key role in shaping the diversity of AsXoo populations [31, 34]. In contrast, AfXoo populations have been less explored, with genetic groups being established through genetic marker schemes [10,11,35] or in some instances associating the genotype with phenotypic reaction to rice isogenic lines containing single *Xa* resistance genes [35–37]. These approaches have been limited in temporal and geographical scope and knowledge of the overall genetic structure of AfXoo on a global scale is still incomplete. While BLB has been established as a threat in Africa [12], it remains unclear how the pathogen originated and dispersed in the continent. The marked differences between the Asian and African phylogroup also make this an interesting case of allopatric speciation and a promising system to study how pathogens adapt to domesticated crops [38]. In this study, we aimed to uncover the evolutionary history and expansion of AfXoo using a population genomics approach. We also addressed the diversity and molecular evolution of AfXoo TALEs in large scale and specific scenarios. Our findings will be key in monitoring AfXoo for successful rice breeding initiatives.

## Results

### Phylogenomics reveal five groups of AfXoo populating West Africa

To investigate the population dynamics of AfXoo in West Africa, we mined the IRD (Institut de Recherche pour le Développement) *Xanthomonas* collection (CIX), which contains *Xoo* strains isolated from rice or rice relatives sampled between the late 1970s to the present (S1 Fig). 333 strains spanning nine African countries were analyzed, while a strain from the Philippines served as an outgroup (S1 Dataset). We first genotyped all of our samples using a Multiple loci VNTR analysis (MLVA) scheme [11], and observed the presence of genetically similar isolates across different parts of Africa (S2 Fig). Some of the strains in our inventory, recently collected in Tanzania and Madagascar [12,39] did not group with other African strains (S2 Fig), phylogenetic analyses showed these strains are part of a group of AsXoo currently spreading in the eastern part of Africa (S3 Fig); these strains are thus considered as part of the AsXoo clade and are discussed elsewhere [11,35].

To have a representative genome set for AfXoo, based on the MLVA results, we selected 56 strains with different haplotypes, geographical origins and/or hosts of isolation for whole-genome long-read sequencing. To also assess diversity at a small geographical scale, a subset of our sequences corresponded to 15 strains isolated in the Bagré region in Burkina Faso, which had been previously shown, based on Southern Blots, to contain variable TALE repertoires [35]. We combined our dataset with other available complete genome sequences from NCBI (S2 Dataset).

In total, 87 AfXoo genomes were analyzed, these strains were monophyletic in a *Xanthomonas oryzae* core gene phylogeny (S3a Fig). An SNP-based maximum likelihood phylogeny analysis together with Bayesian Analysis of Population Structure (BAPS) revealed three monophyletic (BAPS1, 2, and 3) subgroups or populations, a polyphyletic (BAPS4) population, and one strain originating from Mali, isolated from the wild rice, *O. longistaminata,* which formed a separate lineage (BAPS5) (Fig 1a). We validated our BAPS grouping using principal component analysis (PCA) of a SNP matrix, Discriminant Analysis of Principal Components (DAPC) and robustness of the clustering employing Silhouette score, and each approach clearly supports four AfXoo groups present in Africa plus the single BAPS5 strain (S4 Fig). The groups defined by BAPS also largely agree with clustering based on previous [11] and our current MLVA result, but discrepancies arise due to different methods using different genetic distance thresholds to establish groups, as well as confounding factors like the polyphily of BAPS4 (S2 Fig)

**Fig 1:**
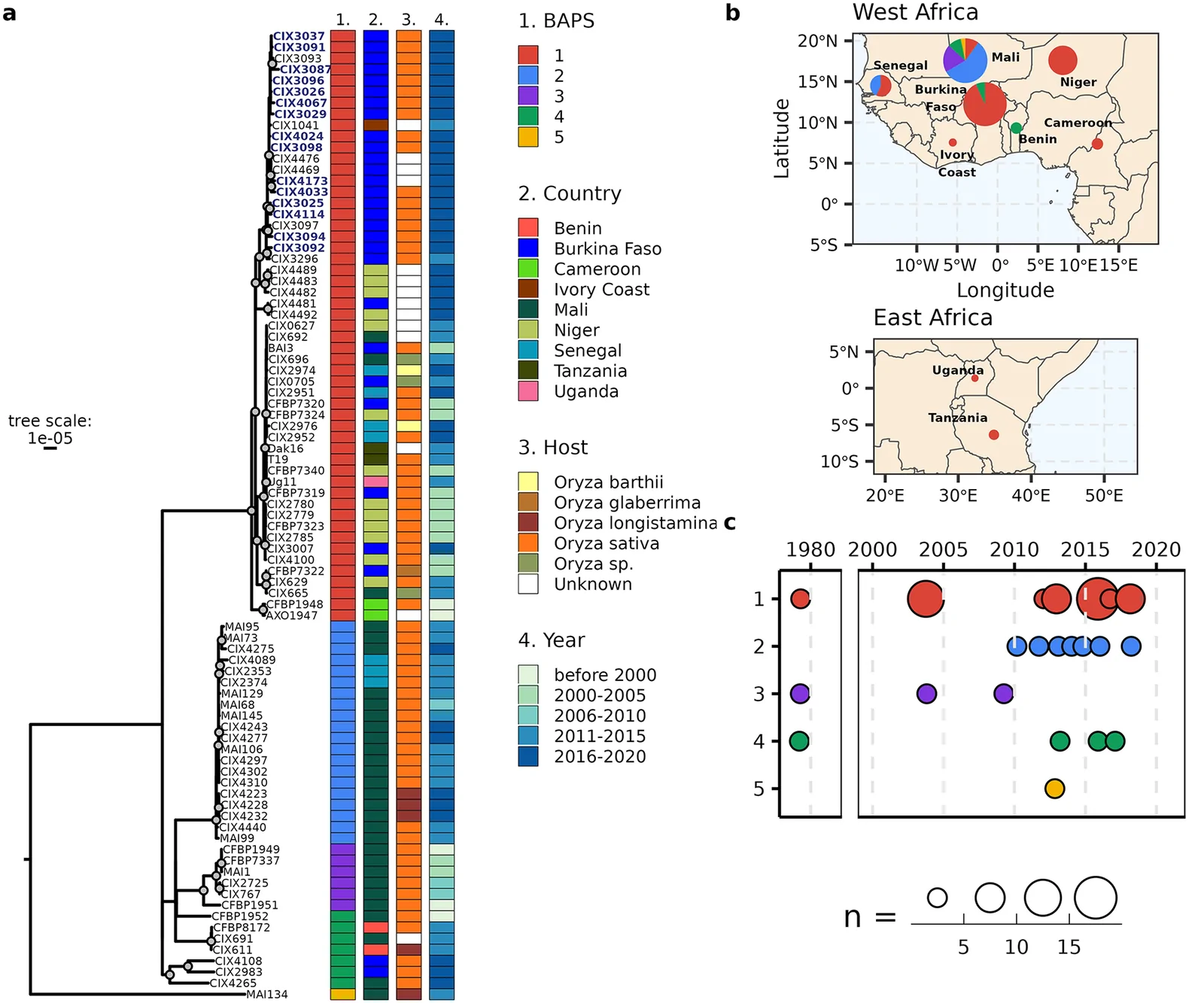
Phylogenetic reconstruction and population structure of AfXoo sampled between 1978 and 2018 in West and East Africa shows five groups. **(a)** Maximum-likelihood whole-genome phylogeny of 87 strains of AfXoo was constructed using 10,653 recombinant-free SNPs with 1000 bootstrap. Gray dots in nodes highlight bootstrap values >= 80%. The population structure was predicted through *rhierBAPS* [40] (Column 1). Information on Country, Host and Year of isolation is shown when available (Columns 2-4). Strain names in dark blue correspond to samples collected in the Bagré region of Burkina Faso between 2016 and 2018, described in Diallo *et al.* [35]. Strain MAI134 was chosen as an outgroup for his tree. **(b)** Spatial distribution of AfXoo population in nine African countries. The size of the pie chart reflects the number of represented strains. The shapefile of Africa was sourced from the Natural Earth project (https://www.naturalearthdata.com/), and parsed into map shapes with the R package rnaturalearth (https://cran.r-project.org/web/packages/rnaturalearth/index.html). **(c)** Temporal distribution of the AfXoo population between 1978 and 2018. The size of the circle corresponds to the total number of strains.

BAPS 1, 2 and 4 are widely distributed across West Africa, with BAPS1 being found in most sampled countries including Tanzania and Uganda, and BAPS3 being restricted to Mali (Fig 1a-1b). Overall, weak phylogeographic structuring was observed in our dataset as isolates from the same region are interspersed across the phylogenetic tree.

Most of the genomes available correspond to strains collected in the 2000s and beyond, and about 5% were collected in the late 1970’s (Fig 1a and 1c). In terms of the distribution of populations over time, BAPS1, 3, and 4 are identified as early as 1979, while BAPS2 seems to be detected mostly after 2010. Most strains were isolated from cultivated Asian rice *Oryza sativa* (71%), yet some strains in BAPS1 were isolated from cultivated African rice *O. glaberrima* or its wild relative *O. barthii*, and some strains from BAPS2, 4 and 5 were isolated from wild rice *O. longistaminata*.

### AfXoo populations include one recombinant group and three clonal groups

We then examined the genetic diversity of each population, their relatedness and recombination among populations using various measures. Because it is represented by a single strain, BAPS5 was excluded from analyses sensitive to sample size. A phylogenetic network tree was constructed using SplitsTree4 [41] and showed a high number of reticulations in BAPS4, and a star-like structure in BAPS1–3 (Fig 2a). This was complemented by structure analysis where BAPS4 was shown to be highly admixed as compared to other groups (S5 Fig). BAPS4 also had a high recombination rate r/m (ratio of the number of SNPs introduced through recombination relative to mutation) of 1.15 and high nucleotide diversity (average π 2.56e-04), compared to BAPS1–3 (r/m range between 0.14-0.57 and average π 2.15e-05-4.13e-05) (Fig 2b-2c). Pairwise post-hoc analysis using Dunn’s test revealed that the r/m ratio between BAPS4 and the rest of the populations (BAPS1: p adj. = 9.20e-08; BAPS2: p adj. = 2.82e-07; BAPS3: p.adj. = 1.62e-03) were statistically different. We further explored the recombination events predicted genome wide in AfXoo populations (S6 Fig). We observed that in BAPS1–3 predicted recombination events are preserved across strains, whereas in BAPS4 they are strain specific (S6a Fig). Our results also showed that BAPS4 acts as both a donor and recipient of genetic alleles for all other populations (S6b Fig). This suggests that BAPS4 is a recombining group and contributed to the diversity of this population.

**Fig 2:**
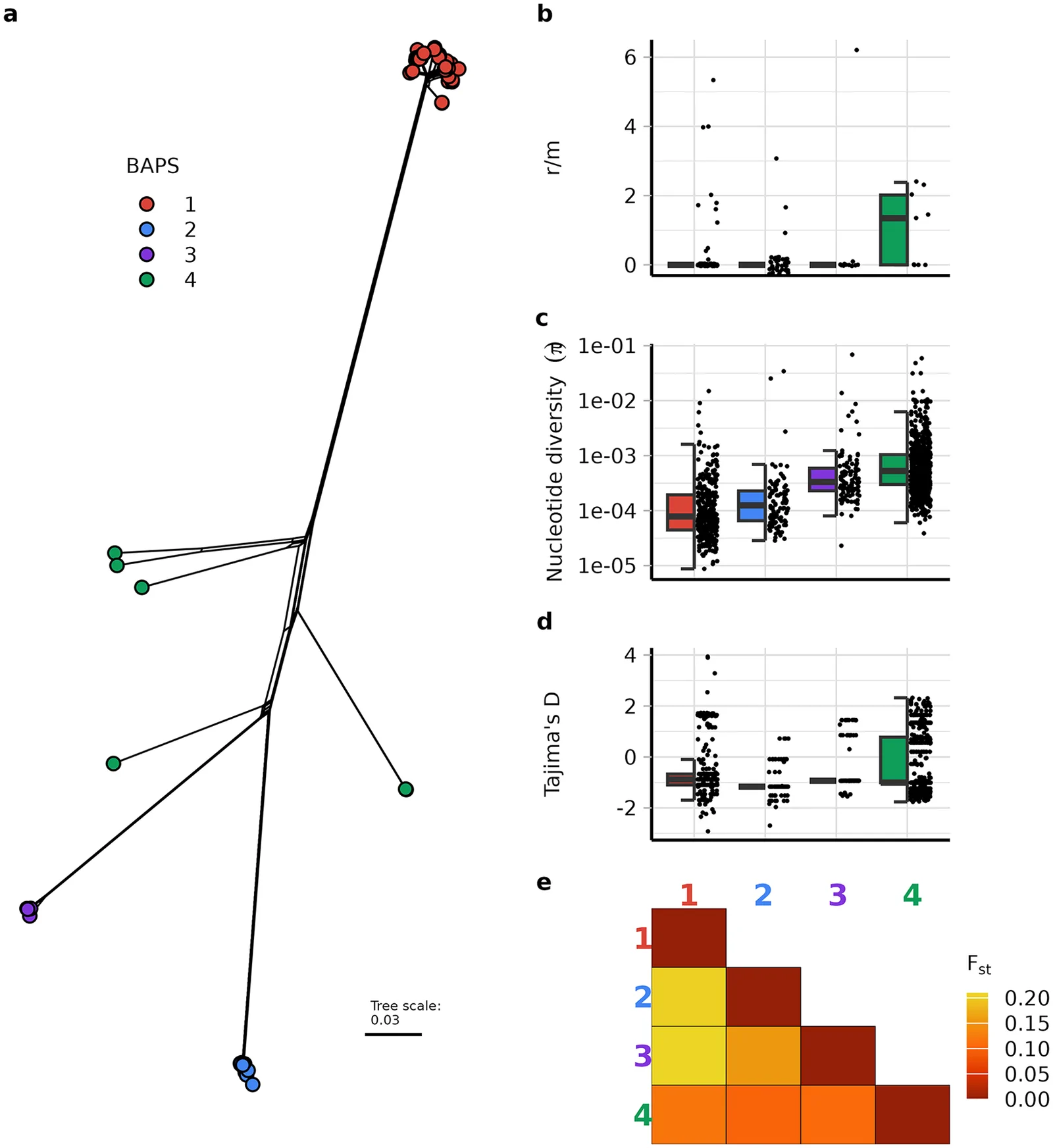
Population dynamics of AfXoo shows three clonal populations and a recombinant population. **(a)** Phylogenetic network showing tight clustering of three populations (BAPS 1-3), and a moderate degree of reticulation in BAPS4. The reticulated phylogeny was generated using SplitsTree4 [42] with recombinant SNPs. **(b)** Boxplot showing the ratio of alleles in the genomic sites that were affected by either recombination or point mutation (r/m). **(c-d)** Nucleotide diversity (π) and Tajima’s D calculated for each AfXoo population. **(e)** Genetic differentiation between AfXoo populations inferred by pairwise fixation indices (F_st_). BAPS5 (strain MAI134) was excluded from these analyses since it was just represented by one genome.

When testing for neutrality, Tajima’s D statistics showed values that were significantly departing from zero for all BAPS (t-test; p < 0.001), suggesting there is strong selection pressure or demographic changes experienced by these populations. All populations showed a negative Tajima’s D (Fig 2d) with median values ranging from -1.16 (BAPS2) to -0.87 (BAPS1), this suggests either a recent expansion or high rates of purifying selection observed in AfXoo populations. Although all the populations have mean negative Tajima’s D values, BAPS 4 has a highly variable distribution across the genome with many positions having a positive Tajima’s D, suggesting that balancing selection might still have a strong effect in particular parts of the genomes. In contrast, BAPS1–3 populations show lower diversity and infrequent recombination, which are hallmarks of a recent clonal population expansion potentially due to a recent bottleneck [43].

We then compared the genetic differentiation between each population by calculating Fst (Fig 2e). BAPS4 showed the lowest Fst values (0.107-0.121) compared to other populations, indicating that this population shares many alleles with all others. This is also reflected in the STRUCTURE result (S5 Fig) where admixture was detected, showing BAPS4 even sharing alleles with BAPS5, the group formed by the single strain MAI134 (S5 Fig). On the other hand, a higher number of unique alleles was observed in BAPS1 compared to the other populations based on Fst (Fig 2e) and pairwise nucleotide diversity (Dxy) (S6 Fig).

Overall, these results indicate that BAPS1–3 are clonal populations differentiated from each other and possibly originating from relative recent bottlenecks, while BAPS4 is a recombinant population that shares alleles with the clonal populations as well as with the more genetically distant BAPS5.

### Molecular clock uncovers a population bottleneck for AfXoo followed by clonal expansion

To establish a timeline for emergence of bacterial blight of rice in Africa by *Xanthomonas oryzae* pv. *oryzae*, we performed a Bayesian molecular clock analysis [42]. The fit of our temporal signal was estimated for datasets with or without BAPS5 by a root-to-tip regression analysis, and then a permutation test was carried out, showing that our heterochronous dataset is only statistically significant for time measured phylogenies when removing BAPS5, thus this strain was again excluded from subsequent analyses (S8a-S8b Fig). Our Bayesian skyline coalescent model (Fig 3a) estimated a mean substitution rate of 4.97e-8 substitutions per site per year (2.18E-8 - 7.98E-8 95% highest posterior density HPD), which is comparable with the AsXoo phylogroup [34] and other *Xanthomonas* species [44,45]. We used phylogenetic molecular clock reconstruction to estimate when the most recent common ancestor (MRCA) of AfXoo groups existed according to allele histories. Our analyses suggests that the MRCA of all the AfXoo studied existed around the year 656 CE (common era) (334 BCE-1351 CE 95%HPD) when *O. glaberrima* was the only cultivated rice in Africa [3], and that the three clonal populations emerged later during the period of adoption of *O. sativa* in West Africa [5]. BAPS1 emerged around 1708 (1489–868 95%HPD), then followed by BAPS3 (1744[1651–1876 95%HPD]) and finally BAPS2 (1946[1892–1986 95%HPD]). We could not estimate an exact date on the origin of BAPS4 because it is polyphyletic, but the molecular clock tree shows it diverged the earliest. To complement the tip-dated tree from BEAST, a molecular clock phylogeny obtained with TreeTime [46] produced similar divergence times (S8c Fig).

**Fig 3:**
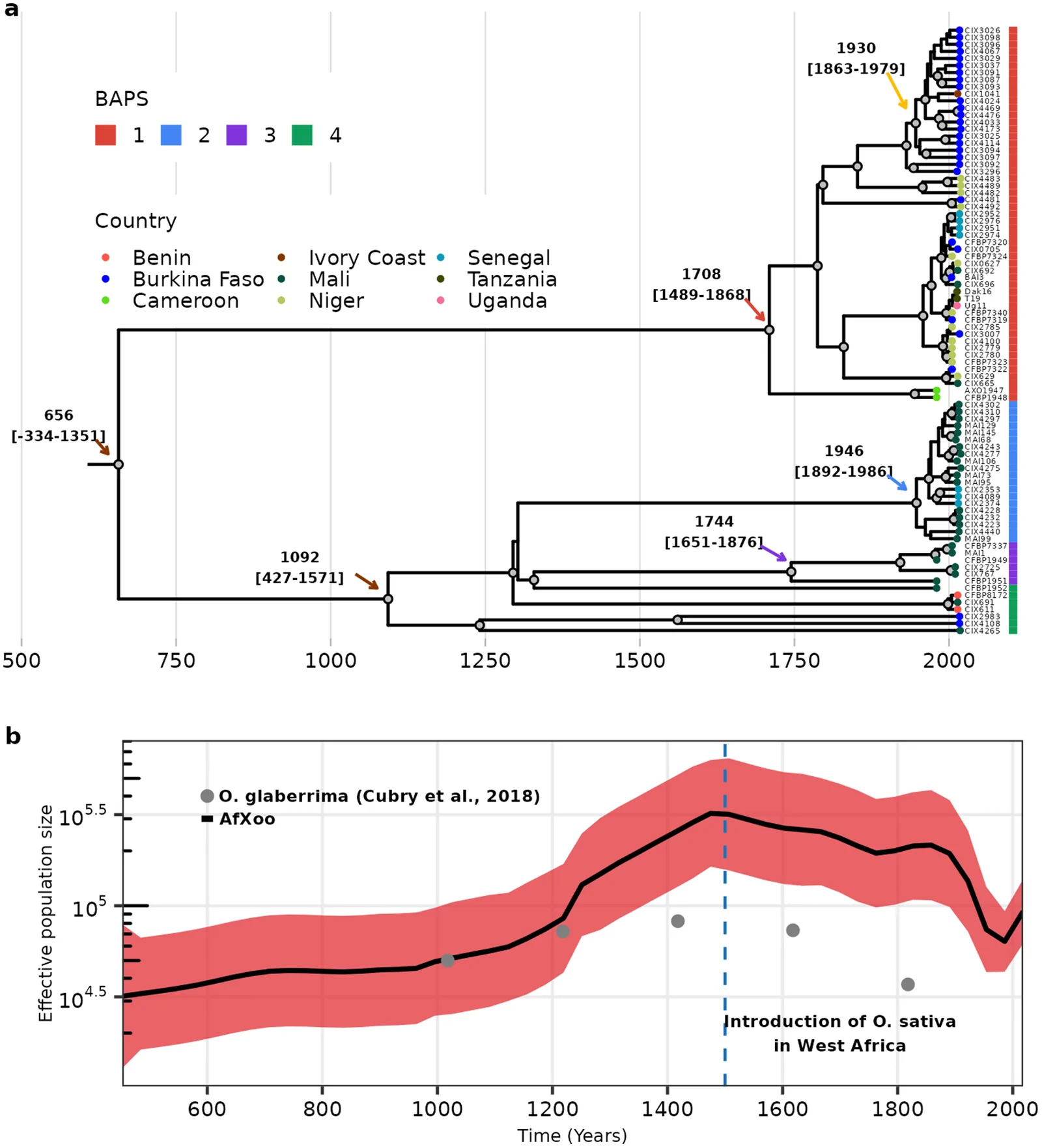
Tip-dating of AfXoo populations shows a genetic bottleneck (a) Maximum clade credibility tree from BEAST analysis [42] was constructed using 4350 recombinant-free SNPs. Mean divergence time is highlighted for the following nodes: the most recent common ancestor for all strains and BAPS2-4 (brown arrows), BAPS3 (violet arrow), BAPS2 (blue arrow), BAPS1 (red arrow) and BAPS1 Bagré strains from Diallo *et al.* [35] (orange arrow). Gray nodes have >95% posterior probability. Years along the x-axis are in the common era (CE). **(b)** A skyline plot of the changes in the effective population size of AfXoo over the years (black line) with 95% confidence interval shown in red. Gray points show estimated effective population size of *O. glaberrima* reported in Cubry et al. [3]. The blue dotted line represents the proposed introduction of *O. sativa* West Africa which in the 16th century [5].

Next, we examined the population dynamics of AfXoo over time according to the coalescent model (Fig 3b). The model initially showed a gradual increase in effective population size between 500–1500 CE. From there (between 1500 to early 1800), AfXoo slowly plateaued and gradually decreased. This period coincides with the introduction of *O. sativa* in West Africa in the early 16th century [5]. The AfXoo population then declined sharply (late 1800 to early 1900) and started to recover around the mid 20th century (Fig 3b). Estimated effective population sizes for *O. glaberrima* based on genomic data also show a decline after the 17th century [3] (Fig 3b).

### The Type III effector repertoire of AfXoo includes TALEs experiencing frequent rearrangements

To understand the virulence repertoire of AfXoo populations, we analyzed the genes encoding for Type III effector (T3E) proteins (S9 Fig). Overall, AfXoo T3Es are highly conserved across populations, with variations mostly occurring between populations and strains within a population often sharing the same alleles. Xop sequences have 1–8 alleles per family. Twelve Xops have highly conserved sequences across all populations, while some Xop alleles are population specific, such as XopI, XopR, XopU and XopZ1 in BAPS1, xopAD and AE in BAPS2, and XopA, XopF1 and XopX in BAPS3.

Next, we annotated Transcription activator like effectors (TALE) content in each strain. In total, 780 TALEs were predicted, and classified into 13 families according to their RVD sequences using AnnoTALE [47] or nine TALE families based on a DisTal phylogeny [48] (S10a Fig; S4 Dataset). The genomic loci of the TALEs are highly syntenic (S10b Fig), and correspond to the 9 families detected using DisTAL and match previously reported groups [15,49,50]. In each locus, TALE sequences have from one to seven alleles based on RVD variation at varying frequency (Fig 4a-4b). Similar to Xops, some TALE sequences are highly conserved like TalC and TalE, while others are highly variable, such as TalF in BAPS1, and some TALE alleles are population specific, for example TalA in BAPS2.

**Fig 4:**
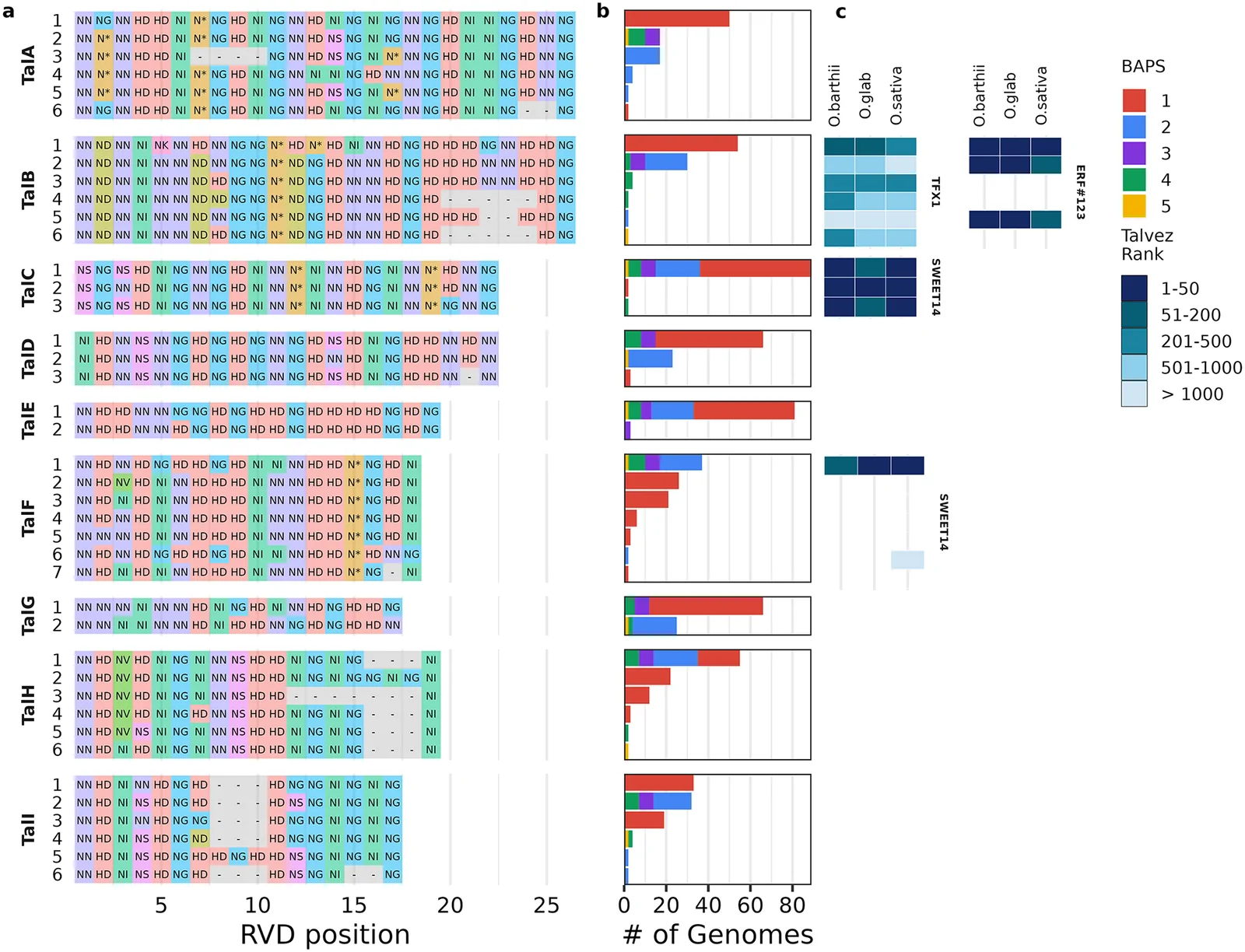
AfXoo strains contain nine diverse TALE families. **(a)** RVD (repeat variable di-residues) variation in central repeat regions of nine AfXoo TALE families/loci. Alleles for each family, as defined by unique RVD sequences, are shown and numbered 1-7 from most to least frequent in the dataset. The color of each block corresponds to a particular RVD sequence. **(b)** Frequencies of TALE family alleles shown in a) colored according to AfXoo population. **(c)** Predicted binding affinity of TALE alleles (indicated as Talvez ranking) to known AfXoo TALE targets with a role in susceptibility. The rankings are based on the prediction score of the EBE (effector binding element) sites of TalB, TalC and TalF in *O. sativa* (Nipponbare v MSU7), and two African rice species, *O. barthii* (IRGC 105608) and *O. glaberrima* (CG14). Predictions were performed with the program Talvez [51] and the complete results are shown in S1 Table.

To understand the molecular evolution of the TALome in AfXoo strains, we examined the variation in repeat and RVD sequences (Fig 4a). Several point mutations were observed in the TALE families, ranging from nine (TalF) to a single (TalC) RVD change. However, we also detected in the RVD alignments changes involving several contiguous repeats, these include indels from 1 to 7 repeats (i.e., alleles 4–6 in TalB), tandem repeat duplications (i.e., allele TalH2) (Fig 4a), and blocks of shared repeats between different TALE loci which might be the result of gene conversion events or convergence towards binding smilar DNA motifs (S11 Fig).

To assess the extent of TALE variation, we compared the genetic distance between TALEs and whole-genome phylogenies. Traditional alignment methods are not well suited to calculate distances between highly repetitive sequences such as TALEs (S12 Fig), so we used the program DisTAL which can calculate distances between repeat arrangements, including also the N and C-termini [48]. For each TALE locus, we calculated the pairwise genetic distances between the TALE alleles of two strains and plotted it against the phylogenetic distance (cophenetic distance) between said strains. As a result, for seven out of nine loci we see a correlation between TALE distance and phylogenetic distance, suggesting these loci change at a constant rate compared to the rest of the genome (Fig 5a)**.** The two exceptions were TalC which is highly conserved, and TalH which on the contrary, shows lots of variation. For most loci however we also detected cases where TALEs from closely related strains were genetically distant (Fig 5a), these cases included variation involving several repeats as detailed above (indels, tandem duplications, repeat swaps) which can happen swiftly due to the repetitive nature of TALEs.

**Fig 5:**
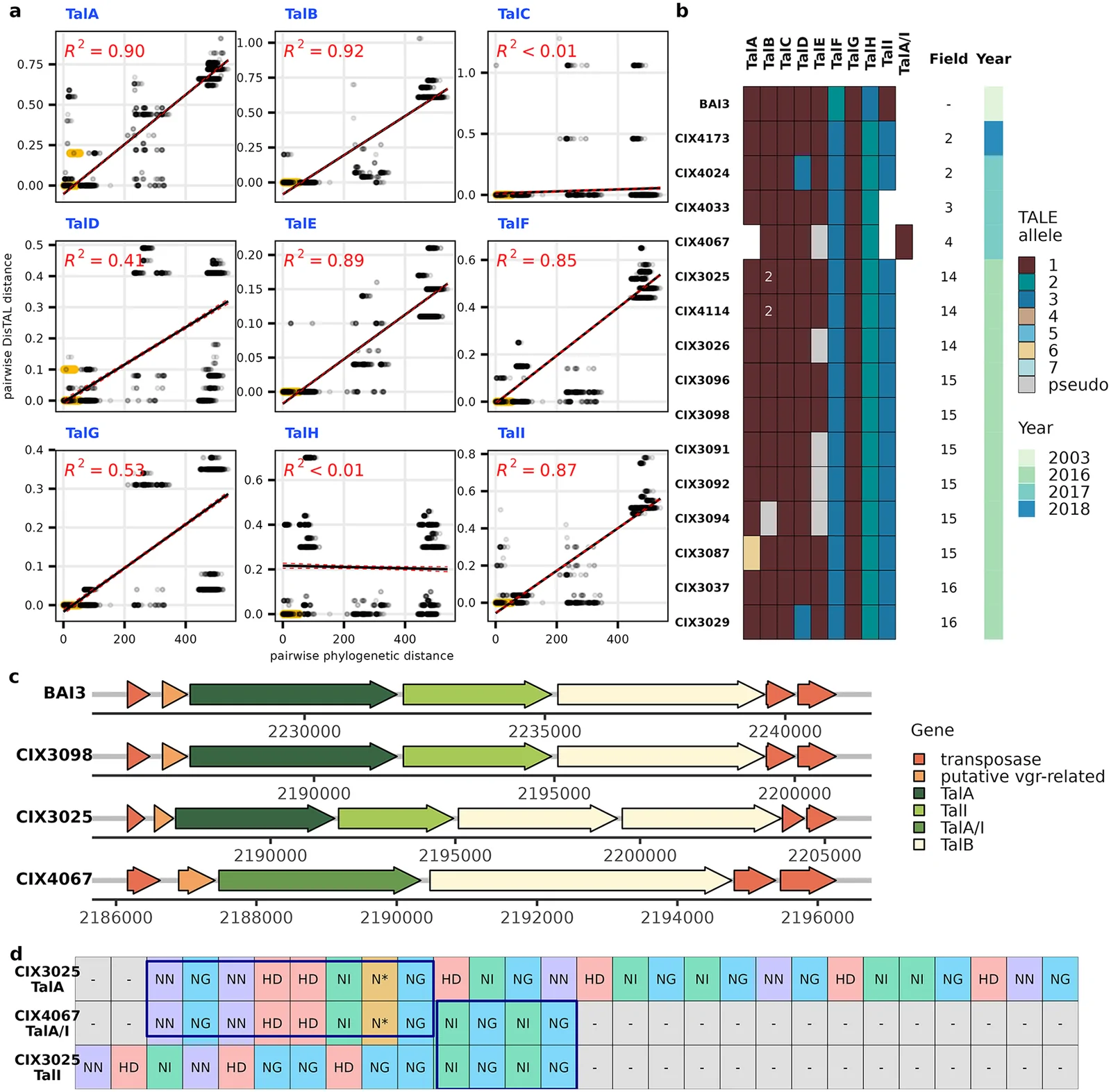
TALE diversity arises between closely related strains through rearrangements. **(a)** Testing for linear relationship between TALE repeat distance and phylogeny in AfXoo. Each point represents two TALE alleles compared in two strains, linear regression analysis was performed on the pairwise DisTAL distance between the alleles [48] versus the pairwise phylogenetic distance (cophenetic distance) between the strains. Points in the upper left part of the graphs are genetically distant TALEs found between closely related strains. Orange points correspond to the Bagré strains highlighted in Fig 1a. The black solid line represents the regression line, and the dashed red lines show the 95% confidence interval. R-squared value is indicated by red text. This analysis does not take into account gain/loss of TALEs **(b)** TALE content of strains sampled in fields in the Bagré Region of Burkina Faso from 2016 to 2018, collection year and field of isolation are shown as detailed in Diallo et al. [35]. TALE alleles are shown colored and numbered according to their frequency in AfXoo and correspond to RVD sequences in Fig 4. Strain BAI3 is shown for comparison. Gray number 2 inside the tile of TalB corresponds to isolates with TalB duplication. **(c)** A gene cluster showing rearrangements in a region covering TalA, TalI and TalB. The gene clusters of BAI3 and CIX3098 are the predominant arrangement in most Bagré samples, while some strains have a duplication of TalB (CIX3025) or experienced a terminal reassortment between TalA and TalI (CIX4067). **(d)** The RVD sequence of TalA/I derived from terminal reassortment of TalA and TalI. Shared blocks of repeats between TALEs are highlighted as blue blocks..  .

We were also interested in examining the variation of TALEs in a relatively small geographic area and in a short span of time, by reexamining TALE variation data from fields in the Bagré region of Burkina Faso [35]. In our previous study we found in a collection of 177 strains collected in Burkina Faso from 2003 to 2018, eight distinct TALome patterns as shown by Restriction Fragment Length Polymorphism (RFLP). To understand the genomic changes behind this variation, we sequenced 15 of these strains collected in nine fields in Bagré between 2016 and 2018 which were regularly prospected; the genome sequences showed TALE content that correlated with the RFLP patterns (S13 Fig).

All the sequenced strains from Bagré belonged to BAPS1 and are estimated to have a common ancestor that existed around the 1930s (Fig 3a), so TALE variation found in this group can be considered recent. As expected from the RFLP, we found rearrangements in TALE sequences between these closely related strains (Fig 5b). These changes include terminal reassortment [56] between TalA and TALI to create a new T3E in strain CIX4067 (Fig 5c and 5d), deletions of two repeats in TalA in strain CIX3296, tandem duplication of the complete TalB sequence in strains CIX3025 and CIX4114, complete deletion of TalI in strain CIX4033, and pseudogenization of TalE due to a frameshift near the Type III secretion signal in five of the strains (S14-S15 Fig). This highlights how TALE sequence diversity can arise in a short time potentially creating phenotypic diversity.

### AfXoo TALE content relates to reported phenotypes

To investigate the virulence capability of AfXoo we compiled phenotype data (S3b Fig; S5 Dataset) from various experiments that used near-isogenic lines (NILs) containing single resistance genes, mainly sourced from *O. sativa* cultivars [10,12,36–38]. These NILs, (named IRBB1, IRBB3, …) carry known resistance genes against *Xanthomonas* (named *Xa1*, *Xa3*, …, respectively) in the genetic background of IR24, a cultivar normally susceptible to AsXoo strains [52]. We observe that populations BAPS3–5 were unable to infect IR24, and accordingly could not infect any IRBB lines (S3b fig). IR24 may carry a resistance gene able to control these AfXoo populations [53]. Some strains from BAPS3–5 cause symptoms in other *O. sativa* backgrounds like Azucena and Komboka and in *O. glaberrima* (S5 Dataset) [12,49].

In contrast, BAPS1 and 2 could infect IR24 and overcome some R genes; *Xa3, Xa4, xa5,* and *Xa7* were able to control AfXoo strains to various degrees. Of particular interest is *Xa7*, as it is an executor R gene that guards SWEET14 [24] and is activated by TALEs AvrXa7 and PthXo3 from AsXoo. While AfXoo strains carry TalC and TalF alleles that induce SWEET14, none of the AfXoo TALEs are predicted to bind to the *Xa7* promoter. It is possible that *Xa7* contributes to resistance to AfXoo independently of its activation by TALEs.

None of the AfXoo genomes studied contained sequences matching interfering TALEs (iTALEs), these are specialized TALEs found in AsXoo with altered repeats and N and C-terminal domains that interfere with recognition of TALEs by the NLR resistant proteins Xa1/Xo1 [54,55]. Accordingly, most AfXoo strains with available data were unable to infect IRBB1 harboring *Xa1* (S3b Fig), with the exception of two strains from Mali that are able to break down *Xa1* resistance in an unknown manner [36]. The presence of iTALEs only in AsXoo may relate to *Xa1/Xo1* being present *O. sativa* and absent in *O. glaberrima* [56].

Next, we predicted the binding affinity of the TALE alleles to the promoters of known rice susceptibility gene targets (Fig 4c; S1 Table). These targets have been shown through experimentation to be up-regulated during AfXoo-*O*. *sativa* interaction [15,57,58]. In addition to *O. sativa*, we used the domesticated African rice *O. glaberrima* and its closest wild relative *O. barthii* in the analysis. *OsSWEET14*-targeting TALEs have disparate rankings. All TalC alleles in all populations are predicted to have high binding affinity (top 200 ranked prediction scores) to *OsSWEET14* and likely all strains rely on its activation for virulence. On the other hand for TalF, only one allele, found in BAPS2, 3 and 4, is predicted to bind to *OsSWEET14* (Fig 4c; S1 Table). The TalF alleles of the strain BAI3 (BAPS1) and MAI168 (BAPS2) were shown to be unable to induce *OsSWEET14* and did not contribute to virulence in plants [15,49]. It is unknown whether other TalF alleles have virulence activity.

Other AfXoo susceptibility genes induced by TalB: *TFX1* (encoding a bZIP transcription factor) and *ERF#123* (a subgroup IXc AP2/ERF transcription factor genes) were predicted to be activated with different affinity according to rice species. All TalB alleles have higher prediction scores for both targets in the genomes of *O. glaberrima* and *O. barthii* compared to *O.* *sativa*, but it is unknown whether these targets are indeed activated in these species and whether they contribute to virulence.

More experimental data is needed to further understand the coevolution of AfXoo and African rice, including the identification of TALE targets exclusive to African rice and their role in virulence.

## Discussion

The bacterial blight pathogen endemic in Africa (AfXoo), while belonging to the same species and pathovar, is genetically distinct from the well-known *Xanthomonas oryzae* pv. *oryzae* in Asia (AsXoo) [10,11]. When comparing both phylogroups, AfXoo genomes (mostly restricted to West Africa), are less diverse, exhibit less recombination, and harbor fewer TALEs; In contrast, AsXoo genomes are more diversified, show a higher recombination rate, a large number of insertion sequences, and large TALE repertoires [16,34].

Allopatric speciation in bacteria has been shown as a result of geographical isolation [59] and, in host-associated bacteria, the host can act as an additional isolation factor [60], such that the evolutionary history of some hosts can be traced by studying the pathogen [61]. The extent to which geographical isolation or adaptation to two hosts (*O. sativa* or *O. glaberrima*) can explain the differences between Asian and African *Xoo* is uncertain. In plant pathogens, domestication and monoculture have been proposed to favor allopatric divergence [62,63]. For *Xanthomonas axonopodis* for example, the existing host-specialized pathogenic pathovars have been proposed to emerge recently from broad host range ancestors, with their specialization (ecological isolation) driven by agricultural development [63].

We hypothesize the differences in diversity of the two *Xoo* lineages might be related to the history of domestication of rice in both continents, as *O. sativa* was domesticated earlier (9,000 years ago) and then adopted throughout Asia for thousands of years [64], while in Africa, domestication of *O. glaberrima* was relatively recent (3,000 years ago) [2]. Accordingly, the MRCA for the most ancestral *Xoo* population in Asia was estimated to exist ~6000 years before present [34], and here we estimate the MRCA for the main populations in Africa to have existed ~1400 years ago. The two domesticated species also represent different genetic and resistant landscapes that the pathogen needed to adapt to. Some virulence mechanisms found only in AsXoo might be specific adaptations to resistance in *O. sativa*, like iTALs evolving to break down *Xa1* resistance [54,55], or the different *SWEET*-activating TALEs evolving as a response to different loss-of-susceptibility alleles (Asian strains target three different *SWEET* genes compared to African strains targeting only *SWEET14*) [65]. Likewise, AfXoo might have less diverse virulence mechanisms since fewer resistance mechanisms have been described in *O. glaberrima* [28]*,* and since *O. glaberrima* is genetically less diverse when compared to *O. sativa* [2]. However, this species has also been significantly less studied, and molecular adaptations exclusive to the *O. glaberrima*-AfXoo interaction are yet to be discovered.

An interesting parallel to the divergence between AsXoo and AfXoo is the case of *Xanthomonas oryzae* pv. *oryzicola (Xoc)*, the pathovar causing bacterial leaf streak. Recent MLVA and genome analyses identified three main genetic groups with somewhat discreet geographical patterns (one ‘Asian’ clade, one ‘East African-Asian’ clade, and one ‘West African’ clade) [66]. The differences between these groups are however less dramatic than for *Xoo*, and ancestral state reconstruction suggests all groups originated in Asia, with introductions occurring later in East and West Africa, followed by diversification [66]. The timing of these introductions could not be determined [67], so it is intriguing whether *Xoc* was present in Africa before the introduction of *O. sativa*, and whether *Xoc* evolution has been or not conditioned by interactions with *O. glaberrima*. *Xoc* strains can cause symptoms in *O. glaberrima*, and strains have occasionally been isolated from this species [66]. *Xoc* strains from Africa however, unlike AfXoo, carry iTALEs and can break down *Xa1* resistance [55,67] a potential feature of co-evolution with *O. sativa.*

We also hypothesize a scenario where AfXoo is initially diverse and adapted to infect *O. glaberrima*, then the introduction of *O. sativa* to Africa and the decline of *O. glaberrima* cultivation [4] impacted the diversity of AfXoo, acting as a bottleneck that eventually favored the clonal expansion of the current populations. This is suggested by our coalescent skyline plot model (Fig 3b) which shows two events of expansion of AfXoo interrupted by a bottleneck. We propose that first, the emergence and proliferation of AfXoo in West Africa coincided with the rise of African cultivated rice (*O. glaberima)* [3]. The Malian region is possibly the center of AfXoo diversification since *O. glaberrima* domestication happened in the inner Niger delta [3], and Mali harbors all the populations found; this could however be due to Mali being more heavily sampled. The introduction of Asian cultivated rice *(O. sativa)* in the early 16th century [5], and the subsequent decline of *O. glaberrima* [3], then gradually altered the population demography of AfXoo in the region, and served as a genetic bottleneck, such that the currently circulating populations are those capable of infecting both species. The AfXoo population has since overcome the resistance of *O. sativa* to varying degrees, with outbreaks of bacterial leaf blight in Africa in the late 1970’s and early 80’s [8,68–70]. Malian strains from currently circulating groups have been shown to indeed be able to equally infect both *O. glaberrima* (CG14) and *O. sativa* (cv. Azucena*)*, with some strains even being more virulent in *O. sativa,* but not the opposite [49]*.*

While it is possible that other factors explain the current and past demography of AfXoo (e.g., environmental changes), the two-step adaptation to two domesticated hosts is a compelling hypothesis. Similar population structures involving bottlenecks and clonal expansion have been reported in other pathogens to be associated with host adaptation [43,71], and loss of genetic diversity might be a feature of a pathogen adapting to domesticated crops [38]. Phylogenomic analyses of North American isolates of *Erwinia tracheiphila*, causal agent of bacterial wilt in cucurbits, showed the population is composed of expanding clusters with low genetic heterogeneity that might have emerged to infect cultivated cucumber *Cucumis* spp. after its introduction to the Americas in the early 1500 [72]. And similarly, emerging outbreaks of *Pseudomonas syringae pv. actinidae* infecting kiwifruit are being caused by genetically homogeneous lineages arising from a source population in a process that might be related to the recent domestication (20th century) of this host [73].

Our analysis revealed AfXoo to be composed of five groups circulating in Africa (Fig 1): a genetically distant group represented by one strain (BAPS5), one recombining (BAPS4) and three clonal (BAPS1–3) populations. Tip-dating analysis (Fig 3a) places the emergence of BAPS1–3 all after the introduction of *O. sativa*, while an ancestor of BAPS4 existed as early as 1000 CE (the timing of BAPS5 emergence could not be determined). The clonal populations might have emerged and expanded thanks to their ability to infect *O. sativa*, with BAPS2 and BAPS3 deriving from a BAPS4-like recombinant ancestor, while BAPS1 derived from an even more distant ancestor (Fig 3). This said, our dataset and analyses are biased precisely because sampling has mostly been made in cultivated, symptomatic *O. sativa* (because of its economical importance) with few samples coming from *O. glaberrima* or wild rices. The full diversity of AfXoo is likely yet to be explored and it might encompass populations not associated with cultivated rice fields. The fact that the diverse BAPS4 and the genetically distant strain MAI134 (BAPS5) were found circulating relatively recently, might hint there is a hidden diversity of AfXoo to be sampled.

The T3E repertoire is important for bacterial plant pathogens, contributing to their host specialization [74–77]. In *Xanthomonas oryzae* pathovars, T3E content has been shown to shape the phenotypic diversity with the host [74,78]. While here, the T3E repertoire of AfXoo was found to be generally conserved (S9 Fig) as was previously observed [15,16,49], allelic variation was found specially between populations and in TALEs. We showed that variation within TALE loci in general correlates to the phylogeny, but that dramatic changes can occur between genetically close strains, in a short time span, through rearrangements (Fig 5a). This highlights the adaptive potential/evolvability of TALEs where variation can be generated quickly as a ‘bet-hedging’ strategy, allowing the population to adapt quickly when confronted with resistance in the host [79]. An exception to TALEs being variable was the TalC locus, which induces *SWEET14* and was conserved in all strains suggesting strong selective pressure exists to maintain its SWEET-inducing activity, and possibly additional virulence functions [79,80]. Curiously, AfXoo strains also carry a TalF allele that induces *SWEET14* binding to an alternative site in the promoter [58]. The *SWEET14*-binding allele of TalF is highly conserved in BAPS2–5, whereas in BAPS1 this locus is highly variable and no alleles are predicted to activate *SWEET14* (Fig 4c). It is unknown whether there are any differences in fitness or virulence when strains carry one or two SWEET-inducing TALEs. The convergent targeting of TalF and TalC might evolutionarily be a response to loss-of-susceptibility alleles like *xa41*, a promoter deletion found in certain accessions of *O. barthii* that prevents activation of SWEET14 by TalF [28]. The presence of one or both TALEs in current AfXoo might have been conditioned by ancestral populations encountering this type of resistance.

In this study we attempted to describe the evolutionary history of *Xoo* in Africa using genomic data, and to associate it with the history of rice cultivation in the continent. While the molecular mechanisms of how AfXoo adapted to their hosts, specially *O. glaberrima*, remain to be detailed, our results hint at the influence of crop domestication in the emergence and spread of pathogens, as reported in other systems [45,81]. Understanding the evolutionary history of this group of bacteria is of particular urgency today, given the ongoing epidemics of BLB unfolding rapidly in East African countries (Kenya, Madagascar, Tanzania and Uganda) as a result of introductions of *Xoo* from Asia [12–14]. Our results highlight that the strategies for control of these epidemics need to be tailored for the introduced strains given the genomic differences between AfXoo and AsXoo. We also raise an alarm regarding the risk of rapid evolution of *Xoo*; we show how AfXoo variation in effector content can appear quickly through rearrangements, a risk that might be exacerbated for AsXoo that harbor more TALEs and generally recombine more [34]. Finally, while currently AsXoo is spreading in East Africa and AfXoo remains mostly restricted to West Africa, the prospect of these populations encountering each other and the potential consequences (competition, synergy, replacement or recombination) are concerning.

## Materials and methods

### *Xanthomonas oryzae* pv. *oryzae* dataset collection and bacterial inoculum

IRD (Institut de recherche pour le développement) has been sampling leaves with bacterial leaf blight (BLB) symptoms in Africa since an outbreak occurred in the late 1970s [8], and a total of 333 live cultures (S1 Dataset) are maintained. The majority of the epidemic samples were collected from West Africa between 1979 and 2018, and a few from the BLB outbreak observed in East Africa [12,13]. Each bacterial strain was maintained in a glycerol stock, and was streaked on PSA medium (10g/L peptone, 10g/L sucrose, 1g/L glutamic acid, 16g/L agar) to obtain single colonies. Plates were incubated for 3 days at 28°C. Individual colonies were resuspended in sterile water and then boiled to lyse the bacteria.

### Collecting the phenotypic data

We compiled pathogenicity data for AfXoo infecting rice from various sources [10,12,35–37 49]. Some of these studies involved inoculating AfXoo strains in IR24 (susceptible check for AsXoo phylogroup) and rice near-isogenic lines (NILs) that carry single resistance genes (S5 Dataset). A review paper from Lore *et al*., [52] describes these NILs in detail. These studies measured the lesion length either 14 or 15 days after inoculation, using varying criteria to record symptoms: resistant (lesion length  ≤ 5 cm) and susceptible (lesion length > 5 cm) or resistant < 5 cm, moderately resistant = 5–10 cm, moderately susceptible = 10–15 cm and susceptible > 15 cm. For consistency purposes, we used the format of resistant (R) ≤ 10 cm, susceptible (S) > 10 cm and R/S for conflicting phenotypes from two references, and converted the results (S2 Dataset).

### MLVA genotyping and amplicon sizing and allele assignment

The AfXoo strain collection and PXO99A as a representative of the AsXoo phylogroup were genotyped using the MLVA-16 scheme developed for *Xanthomonas oryzae* [11]. Two loci (G58 and G88) were excluded from the analyses because of amplification issues. The multiplex PCR reactions were performed directly on the bacterial lysates. Four reactions of multiplex PCR (corresponding to four different mixtures) were performed with fluorescent primer pairs, using the QIAGEN Multiplex PCR kit (Qiagen, Courtaboeuf, France). The amplification conditions for mixes 1 and 2 were 35 cycles of 94°C for 30 s, annealing temperature of 60°C for 1 min 30 s and 72°C for 1 min. For mixes 3 and 4, the conditions were 25 cycles with an annealing temperature of 64°C. PCR products were diluted in Hi-Di formamide mixed with GeneScan 500 or 600 LIZ dye Size Standard (Thermofisher Scientific). Capillary electrophoresis was performed on an ABI 3500 XL Genetic Analyzer (Applied Biosystems) at the GenSeq platform in Montpellier, France.

The fluorescent peaks obtained for each locus and strain were sized using GeneMapper version 4.0 (Applied Biosystems, Life Technologies, Carlsbad, CA). Peaks corresponding to fragments sizes below the combined size of the flanking regions of the specified VNTR were deleted. The same software was used to assign alleles to each fragment size. When necessary, the size was rounded up to the nearest integer and corresponding allele. Minimum spanning trees were generated using goeBURST [82] in PHYLOViZ 2.0 [83].

### Genome sequencing, genome assembly and annotation

Combining our MLVA results with those of Diallo et al. [35] focusing on BLB in Burkina Faso, 59 strains were selected for genome sequencing using long reads either from Oxford Nanopore technology (ONT) or SMRT technology. First, genomic DNA was isolated applying the Qiagen Genomic-tip 100/G following the manufacturer protocol. For ONT sequencing, the genomic DNA was then subjected to multiplex library construction through an ONT rapid library preparation kit, and sequenced using the MinION 4ONT device (Oxford, UK). Guppy v6.0.1 was used to perform read basecalling with MIN_QSCORE >= 9. ONT read adapters and barcodes were removed with porechop [84]. As for the PacBio sequenced data, they were sent to Icahn Institute for Genomics and Multiscale Biology (New York, NY, United States). Then, the newly sequenced reads were used to carry out de-novo assembly of each strain adopting the CulebrONT workflow v2.0.1 [85]. The pipeline uses FLYE v2.9-b1768 [86] and Miniasm v0.3-r179 for the assembly and contigs were then polished with Medaka v1.4.1. The quality of the newly assembled genomes was assessed by BUSCO v5.5.0 [87] using the Xanthomonadales Odb10 datasets. Complete or nearly complete genomes were obtained with one to two contigs, and some harboring plasmids, as previously observed [88]. Additional AfXoo and other *Xanthomonas oryzae* genomes were downloaded from NCBI. The list of genomes used in this study, and the corresponding metadata are provided in S2 and S3 Dataset. Finally, all genomes were annotated using Bakta v1.9.2 [89].

### Phylogenetic reconstruction and recombination

For phylogenetic reconstruction, a maximum likelihood (ML) tree was produced for *Xanthomonas oryzae* species. To build a multiple core gene alignment, genes were first clustered into orthologous groups using Panaroo v1.2.10 [90] in strict mode, paralog clusters were merged, and invalid annotated genes were removed. SNPs were extracted from the core gene alignment using snp-sites v2.5.1 [91], and used to create a ML phylogeny by IQtree v2.2.2.6 [92]. This SNP extraction method was performed since *Xanthomonas oryzae* phylogroups are highly divergent, and the pangenome captures more of the diversity. Parameters for phylogeny construction were set with the ultrafast bootstrap at 1000, specifying the substitution model GTR + I + Γ, and the *Xanthomonas oryzae* strain from the US, X11-5A, as the outgroup. To estimate accurate branch lengths, invariant site information was added, and was obtained from snp-sites.

The phylogeny for AfXoo was carried out by first conducting a multiple alignment to acquire the core genome and extract single nucleotide polymorphism (SNP) with BAI3 as the reference from the core alignment using parsnp v1.5.6 which is included in the Harvest suite tools [93]. This SNP extraction method is mostly used for closely related strains and it also captures the intergenic regions which Panaroo cannot. The resulting core genome was approximately 3.93 (10,653 SNPs) and 3.89 (4350 SNPs) Mb with or without BAPS5, respectively. The core genome alignment was utilized for detecting genomic regions under recombination with Gubbins v3.3.1 [94], and an ML tree was built through RAxML v8.2.12 [95]. The flow of the detected recombination events between populations were characterized by finding the closest source of the recombination (donor) through a pairwise genetic dissimilarity function (*dist.gene*) in the R package *ape* [96]. Strains from other populations that have at least zero to three nucleotide differences in the sequence with the recombination tract are predicted to be the potential donor population. The AfXoo network phylogeny was constructed as an unrooted phylogenetic network using the Neighbor Net method in SplitsTree4 v4.19.2 [41] using the core genome SNP matrix with recombination.

To construct the Bayesian molecular clock trees, an initial analysis to test the suitability of our heterochronous data was performed. Two tests were executed, and these are building root-to-tip regression analysis [97] and a random permutation of sampling dates [98] for datasets that either included or excluded BAPS5. From these tests, our data showed a significant positive correlation for a dataset without BAPS5 (S8a-S8b Fig). BEAST v1.10.5 [42] was used to conduct the temporal phylogeny under a strict or lognormal molecular clock with either constant or skyline coalescent models with recombination-free SNPs. In addition, the GTR + Γ + I substitution model was applied and taking into account the number of invariant sites. Each combination was repeated five times with 100 million Bayesian Markov chain Monte Carlo (MCMC) steps, and the posterior was sampled every 10,000th iteration. Each model combination was taken as input using Tracer v1.7 to check for proper convergence and to inspect the effective sample size (>= 200). The logs and trees of the models were combined after discarding 10% as burn-in using LogCombiner v1.10.5. The final phylogeny was summarized by a maximum clade credibility tree TreeAnnotator v1.10.5 removing 10% as burn-in, and reporting median heights. To complement the temporal phylogeny obtained from BEAST, the ML tree without BAPS5 and the temporal data described above was used as input to infer molecular clock via TreeTime v0.11.1 [46]. The detailed parameters for TreeTime are as follows: --stochastic-resolve (resolving polytomies), --coalescent skyline (estimate changes in the population) --gtr infer (GTR model) --confidence (divergence time confidence interval) and --max-iter 1000 (optimizing the best tree search).

In addition, computation for changes in effective population size were calculated from the results of BEAST and TreeTime. Estimates of population size dynamics were calculated using a skyline coalescent model available in both programs. All phylogenies generated here were imported and stored with the R package *treeio* [99] and visualized by *ggtree* [100] or *tanggle* [101] for the network phylogeny.

### Population structure and population genetic analysis

To infer the genetic relationships in AfXoo, we used the SNP data extracted from parsnp. The predictions were obtained from the R package *rhierBAPS* [40] which implements hierarchical Bayesian Analysis of Population Structure (BAPS) with two levels of clustering. The initial number of groups obtained was five and at the second level was fifteen. Principal component analysis (PCA) was also performed on a binary SNP matrix using the function *prcomp* in R. In addition to the *rhier*BAPS result, genetic structure was also predicted using Discriminant Analysis of Principal Components (DAPC) [102] from the R package *adegenet*. The number of clusters (K) was determined de novo, and the optimal number of principal components to retain was determined using the function *optim.a.score* command. The optimal K was observed to be at four clusters based on both the lowest Bayesian Information Criterion (BIC) value and from the highest silhouette score computed from the *cluster* R package (https://cran.r-project.org/web/packages/cluster/index.html) (S4b-S4c Fig). The population structure was also inferred using STRUCTURE v2.3.4 [103] from K2-6 for ten independent runs, and the results were compiled in the R package *pophelper* [104].

Genetic diversity analysis was explored between and within AfXoo groups. Genes were first clustered into orthologous groups using Panaroo with the parameters mentioned above. The result showed a total of 4959 orthogroups, of which at 82% (4069 orthologous genes) are part of the core genes (>= 95% of the total strain), and the remaining 13% (890 orthologous genes) are the accessory/dispensable genes. The orthogroups were then aligned using Mafft v7.522 [105]. To calculate population genetic statistics, the core genes were used in the R package *PopGenome* [106]. To estimate diversity and genetic differentiation within and between AfXoo groups, nucleotide diversity (π), the pairwise fixation index (F_ST_), and absolute divergence (D_XY_) were estimated. Demographic changes in each AfXoo group were evaluated by Tajima’s D neutrality statistics. Statistically significant differences in each analysis were determined by the Wilcoxon signed-rank test.

### Distribution and evolution of Type III effectors repertoire

The type III effector repertoire of *Xanthomonas* species is divided into two groups Xops (Xanthomonas outer proteins) and TALEs (Transcription activator-like effectors) [19]. The presence of Xops in the AfXoo was searched from the orthogroups using blastx with at least 90% identity and coverage and an e-value of 1e-30. The *Xop* genes were extracted from a community curated Xanthomonas database [107]. Next, TALEs were identified and classified into families using AnnoTALE v1.5 [47] (S4 Dataset). A neighbor-joining (NJ) phylogeny based on TALE repeat distances was constructed using DisTAL v1.1 which is part of the QueTAL suite [48]. The NJ tree was visualized using *ggtree*. The observed TALE families from the DisTAL tree in AfXoo were congruent with previous studies [15].For TALE target prediction, we used the unique RVD sequences of each TALE family against the promoter region of annotated genes in *O. sativa* (Nipponbare v. MSU7), *O. glaberrima* (CG14, GCA_000147395.3), and *O. barthii* (IRGC 105608, GCA_000182155.2) at 1000 bp before the translation start site using Talvez v.3.1 [51]. Only the known TALE-rice targets with a role in susceptibility were shown: *SWEET14*, *TFX1*, and *ERF#123* [15,58] (S1 Table). In addition, we also included the *Xa7* promoter sequence in the search for EBE targets.

For the regression analysis, we computed the pairwise TALE sequences similarity by using the distance matrix file from the result of DisTAL, which computes similarity based on repeat arrays, and by aligning the amino acid version of the TALE repeats using DECIPHER v3 first [108], and then distance similarity was measured through Levenshtein method through the R package *pwalign* (https://bioconductor.org/packages/pwalign). For pairwise branch length distance of each strain pair, we calculated the cophenetic distance using the R package *ape* [96].

To get a glimpse of the TALE evolution in the field, we complemented the predicted TALE with the TALome information from RFLP Analysis [35]. Briefly, the field strains from Bagré, Burkina Faso collected in 2016–2018 were compared for their TALE content, and the results from the previous study showed the presence of six TALome haplotypes. In our case, each TALE family RVD sequence from the six haplotype were compared for variation. In the case of TalE, we aligned the N-terminal of both nucleotide and amino acid sequences with Mafft and visualized them with *ggmsa* [109].

## Supporting information

S1 Fig Spatial and temporal frequency of AfXoo strains (n = 333) per country characterized by VNTR analysis in this study. The countries of origin are highlighted on the map using the same color code. The donut chart shows the proportion of the strains per country, while the bar plot at the top-right corner highlights the sampling date of strains from each country. These strains were characterized using VNTR analysis, and a subset of 39 strains were whole genome sequenced. Additional 17 strains were selected for sequencing based on geography, but were not typed with VNTR. The shapefile of Africa was sourced from the Natural Earth project (https://www.naturalearthdata.com/), and parsed into map shapes by the R package *maps* (https://cran.r-project.org/web/packages/maps/index.html).
(TIF)

S2 Fig Minimum spanning tree (MST) of AfXoo based on multiple locus VNTR haplotypes. The number in each circle corresponds to the identifier of an individual haplotype. Numbers above the links between haplotypes indicate the number of polymorphic VNTR loci that distinguish the haplotypes. Black links connect Single Locus Variants (SLV), while gray links connect haplotypes differing by two or three VNTR loci. Haplotypes that differ by more than three loci are not linked together. The Discriminant analysis of principal (DAPC) clusters are presented. The colors of each circle display information about the isolates grouped into a haplotype and their country of origin.
(TIF)

S3 Fig Phylogenetic relationship and population structure of AfXoo. **a.** Maximum- likelihood (ML) phylogeny showing different *Xanthomonas oryzae* (n = 265) phylogroups. The ML tree shows that AfXoo is a distinct monophyletic group, and is genetically different from AsXoo which also causes bacterial blight. The gray dots in some nodes of the phylogeny indicate bootstrap values greater than 95. **b.** An ML tree constructed using 10,653 recombinant-free SNPs with 1000 bootstrap. The population structure was predicted through *rhierBAPS* (column 1) [40], and DAPC analysis (column 2 and 3) from MLVA datasets taken from Poulin et al., 2015, and the one performed in this paper, respectively. The final columns show the virulence reactions (S: susceptible, R: resistant or R/S: neither resistant or susceptible) to a set of near-isogenic lines carrying single resistance genes (*Xa*) from collated data (S2 Dataset). The IR24 rice line is the standard for checking susceptibility to AsXoo.
(TIF)

S4 Fig Population structure analysis strongly supports the existence of five clusters in AfXoo. **a.** Principal component analysis of the SNP matrix reveals five groupings. **b.** DAPC analysis shows five as the most probable cluster based on BIC values. **c.** The mean silhouette score was the highest at k = 5.
(TIF)

S5 Fig Structure v2.3.4 results showing genetic groups from K = 2–5 genetic groups. The color bars represent the probability of membership to a cluster. The Structure results were summarized in the R package *pophelper* (Francis, 2017) [104].
(TIF)

S6 Fig Recombination events detected in AfXoo. **a.** The plot illustrates the 234 predicted recombination events in the core genome of AfXoo as identified by Gubbins [94], shown as red dotted lines. The gray dotted lines show the location of type III effectors that are inside the recombination tract. The colored numbers displayed at the left-side of the plot are predicted BAPS groupings. **b.** Heatmap showing the flow of the recombination events from the donor to the recipient AfXoo population. The flow of recombination was determined based on a pairwise distance that shows the number of sequence differences between the recombination tract and sequences from other populations. The color in the x- and y-axis indicates BAPS groupings.
(TIF)

S7 Fig Genome-wide pairwise nucleotide diversity within and between AfXoo populations. The results shown diagonally are within genome-wide nucleotide diversity, while the rest are between pairwise nucleotide genetic variation. The line shows the average pairwise nucleotide diversity between and within populations.
(TIF)

S8 Fig Testing for temporal signal and molecular tip-dating in AfXoo. **a-b.** Linear regression analysis of root-to-tip divergence versus sampling dates for with MAI134 (BAPS5) or without. The strain MAI134 is highlighted in yellow. The red dotted lines show the 95% confidence interval. **c.** Inferring a molecular clock phylogeny using TreeTime [46] produced results that overlapped with those in Fig 3a. The colors feature the predicted emergence of AfXoo (black) and selected populations (BAPS1 (red), BAPS2 (blue), BAPS3 (violet).
(TIF)

S9 Fig Clustering of AfXoo strains based on Type III effector alleles. There are 9 main and one novel (TalA/I) Transcription activator-like-effectors (TALEs) and 27 Xanthomonas outer proteins (Xop) that can be potentially present in AfXoo strains. The TALE alleles are ranked from most (1) to least common (6). As for Xop, alleles that are shown to be frequent are classified as 1 and unique alleles are shown as singletons. The clustering is based on an average hierarchical clustering approach using a binary distance. Both effector types were analyzed using the amino acid sequences.
(TIF)

S10 Fig Distribution of TALEs families in AfXoo populations. **a.** Neighbor-joining tree of TALEs based on distance from alignment matrix of repeat sequences in the tool DisTAL [48]. Highlighted in the phylogeny are AnnoTALE [47] clusters, and the TALE family described in Tran et al., 2018 [15]. **b.** Graphical visualization of TALEs loci in AfXoo genomes. The number inside the parentheses indicates the number of strains with that particular TALE locus conformation. The genome CIX1041 was excluded in this analysis because the genome is not complete (13 contigs) even though all the nine TALEs are present.
(TIF)

S11 Fig Shared blocks of repeats were identified between TalF and other TALEs loci. The graph shows blocks of repeats shared between different loci, suggesting rearrangements, potentially resulting from gene conversion events in some parts of the TALE repeat region as highlighted in the red block.
(TIF)

S12 Fig Testing for linear relationship between TALE repeats and phylogeny in AfXoo. Linear regression analysis was performed on the pairwise sequence variation versus pairwise phylogenetic distance. The difference in TALE repeats sequences were calculated through Levenshtein distance, and the pairwise phylogenetic distance by cophenetic distance. The black solid line represents the regression line, and the dashed red lines show the 95% confidence interval. The red text inside the plot is the R-squared value. The orange points correspond to the pairwise data of the Bagré strains that are described in Figure 1a and Figure 5b.
(TIF)

S13 Fig Distribution of TALome patterns in AfXoo strains from Bagré, Burkina Faso using RFLP and whole genome sequencing. The TALome patterns obtained from the sequencing data. The x-axis shows the strains from Bagré and the y-axis is the size (bp) of the TALE. The color of the strains reflects the observed TALome pattern shown from Diallo et al. [35].
(TIF)

S14 Fig The amino acid alignment shows a shorter version of the type III secretion signal of pseudogenized TalE due to a frameshift mutation. (TIF)

S15 Fig An insertion of T at position 848 inside the TalE near the type III secretion signal nucleotide sequence has caused a frame shift mutation. The insertion is shown with an arrow and “in/del” sign.
(TIF)

S1 Table Talvez results from known TALE targets for each TALE allele and *Oryza* species. (XLSX)

S1 Dataset List of the *Xanthomonas oryzae* pv. *oryzae* strains used in this study for MLVA. (XLSX)

S2 Dataset General information of all the African *Xanthomonas oryzae* pv. *oryzae* (AfXoo) used in this study for genomics. (XLSX)

S3 Dataset General information of the other *Xanthomonas oryzae* strains used in this study. (XLSX)

S4 Dataset The list of Transcription Activator-Like Effectors (TALEs) annotated from 87 African *Xanthomonas oryzae* pv. *oryzae* (AfXoo) genomes. (XLSX)

S5 Dataset The list of the original African Xanthomonas pv. oryzae (AfXoo) phenotype data from different references. (XLSX)
