# Supplementary material for: Two domesticated species of rice shaped the population structure of Xanthomonas oryzae pv. oryzae in Africa
Source: PLoS Pathog. 2026 Sep 17;22(9):e1014015. doi: 10.1371/journal.ppat.1014015 (PMC13619019; doi:10.1371/journal.ppat.1014015)
Supplement: Supplementary file 1 [file ppat.1014015.s022.docx]

Dear editor,

We are submitting the revised version of our manuscript titled ‘**Two domesticated species of rice shaped the population structure of *Xanthomonas oryzae*** **pv. *oryzae* in Africa’** for consideration for publication as a Research Article.

We appreciate the comments the editor and reviewers proposed to improve the manuscript and we have made our best to address them, we agree that in the original version of our manuscript tended to mix results with hypotheses. We have extensively modified the manuscript so the result section states better what the data shows and the discussion section shows what we hypothesize from the data.

We also performed additional analyses related to recombination as suggested by the reviewers, we have clarified the analyses regarding genomes from the Bagre region of Burkina Faso and integrated them better in the genome, and we have performed additional analyses to assess how TALE effector variation arises.

We were unable to perform experimental assays suggested by the reviewers, these we consider will indeed be valuable and will be carried out but we consider them to be beyond the scope of this manuscript and will be the subject of future manuscripts.

We have also addressed changes requested by the editorial team including clarifying that the maps used in Figure 1 and SFig 1 were obtained from the public domain source Natural Earth, and we removed a blot image from S13 Fig that was originally included for illustration purposes and for which the raw file was not available.

We thank the reviewers and editor for the care they put in evaluating the manuscript. We believe the current version of the manuscript better reflects the analyses we have made on the population and evolutionary genomics of African *Xoo* and will be of value, not only for the rice-*Xanthomonas* research community, but more broadly to understand the evolution of pathogens in agroecosystems.

We next provide detailed answers to the reviewers' concerns.

Cordially.

Alvaro L Perez-Quintero	Sebastien Cunnac

**Response to reviewers**

# Part I - Summary

Reviewer #1: This manuscript offers a significant contribution to understanding Xoo diversity and the evolution of a rice pathogen in Africa. The addition of the whole-genome sequencing AfXoo dataset is a novel contribution. However, the paper suffers from speculative claims about what their data shows. In addition, the focus is currently limited, and could benefit from discussion of literature outside rice pathogens and Xoo to general impacts of allopatric pathogen evolution, or host introductions on pathogen evolution.

Response: Thank you for this suggestion, we have reduced speculation in the results and we have now added sections in the discussion addressing the broader significance of our findings and comparing it to other works. Some of these changes are in more detail in responses to Part II.

We have also added comparisons to other systems, relevant to our hypotheses on host adaptation. When discussing the impact the introduction of *O. sativa* had in the African Xoo population, we state:

PAGE 17, LINES 429-440. ‘While it is possible that other factors explain the current and past demography of AfXoo, the two-step adaptation to two domesticated hosts is a compelling hypothesis. Similar population structures involving bottlenecks and clonal expansion have been reported in other pathogens to be associated with host adaptation (41,70), and loss of genetic diversity might be a feature of a pathogen adapting to domesticated crops [38]. Phylogenomic analyses of North American isolates *Erwinia tracheiphila*, causal agent of bacterial wilt in cucurbits, showed the population is composed of expanding clusters with low genetic heterogeneity that might have emerged to infect cultivated cucumber *Cucumis* spp. after its introduction to the Americas in the early 1500 [71]. And similarly, emerging outbreaks of *Pseudomonas syringae pv. actinidae* infecting kiwifruit are being caused by genetically homogeneous lineages arising from a source population in a process that might be related to the recent domestication (20th century) of this host [72].’

The claim about distinct lineages was well supported with NVTR and WGS data. However, there were repeated claims that clonal lineages BAPS 1, 2, and 3 were derived from recombinant lineage 4. Based on the phylogenetic trees presented (esp. Figure 3B), baps 1 is a sister lineage to baps 2, 3, and 4. I do not think the phylogenetic data shows any evidence of the derived population claim for lineage 1 specifically.

Response: Thank you for bringing this to our attention, we agree and have modified statements regarding the relations between the clades throughout.

We summarize the population structure now as follows: PAGE 9, LINES 223-226. ‘Overall, these results indicate that BAPS1-3 are clonal populations differentiated from each other and possibly originating from relative recent bottlenecks, while BAPS4 is a recombinant population that shares alleles with the clonal populations as well as with the more genetically distant BAPS5.’

And in the discussion the proposed scenario of emergence for each group is as follows PAGE 18, LINES 441-448. ‘Our analysis revealed AfXoo to be composed of five groups circulating in Africa (Fig 1), a genetically distant group represented by one strain (BAPS5), one recombining (BAPS4) and three clonal (BAPS1-3) populations. Tip-dating analysis (Fig 3a) places the emergence of BAPS1-3 all after the introduction of *O. sativa*, while an ancestor of BAPS4 existed as early as 1000 CE (the timing of BAPS5 emergence could not be determined). We can imagine a scenario where the clonal populations emerge and expanded thanks to their ability to infect *O. sativa*, with BAPS2 and BAPS3 deriving from a BAPS4-like recombinant ancestor, while BAPS1 derived from an even more distant ancestor (Fig 3).’

The diversity in TALE and Xop genes were well presented and documented, but I found claims about recombination in TALE sequences to be unsubstantiated (no actual tests for recombination).

Response: We attempted to use methods for recombination but these are not suitable for TALE effectors, as a solution we calculated TALE distances using DisTAL (Perez-Quintero *et al.*, 2015) and identified instances of TALE pairs with high variation between closely related strains, which could be the result of recombination. A similar strategy has been applied to estimate recombination in TALEs elsewhere (Erkes *et al.*, 2017). Throughout the paper we also removed claims of recombination when they were unsubstantiated.

The section dealing with TALE variation now reads as follows PAGE 11-12, LINES 286-299: ‘To assess the extent of TALE variation, we compared the genetic distance between TALEs and whole-genome phylogenies. Traditional alignment methods are not well suited to calculate distances between highly repetitive sequences such as TALEs (S12 Fig), so we used the program DisTAL which can calculate distances between repeat arrangements, including also the N and C-termini [47]. For each TALE locus, we calculated the pairwise genetic distances between the TALE alleles of two strains and plotted it against the phylogenetic distance (cophenetic distance) between said strains. As a result, for seven out of nine loci we see a correlation between TALE distance and phylogenetic distance, suggesting these loci change at a constant rate compared to the rest of the genome (Fig 5a). The two exceptions were TalC which is highly conserved, and TalH which on the contrary, shows lots of variation. For most loci however we also detected cases where TALEs from closely related strains were genetically distant (Fig 5a), these cases included variation involving several repeats as detailed above (indels, tandem repeats, repeat swaps) which can happen swiftly due to the repetitive nature of TALEs.’

My major issue with this paper was the lack of evidence demonstrating a link between the rice cultivars present in West Africa and pathogen evolution. Throughout the results section, there were speculations about the introduction of O. sativa and pathogen host specialization from native to an introduced rice species. I do not think there is any concrete data supporting this, and these speculations were overstated and presented as a main finding of the manuscript.

Response: We have modified the results section to tone down the speculative aspects, and now most aspects dealing with the effect of the introduction of O. sativa are in the discussion. However, we do think it is important for context for the reader to compare the dates we obtained from tip-dating and our coalescent model, against the documented introduction of *O. sativa* and the decline of *O. glaberrima* based on genomic data (Cubry *et al.*, 2018). Figure 3 now includes points from estimated effective population size for *O. glaberrima* as reported in Cubry *et al*. (2018) and the introduction date of *O. sativa.*

The section in the results comparing the inferred effective population size of AfXoo against that of *O. glaberrima* and *O. sativa* now reads as follows PAGE 10, LINES 249-256.

‘Next, we examined the population dynamics of AfXoo over time according to the coalescent model (Fig 3b). The model initially showed a gradual increase in effective population size between 500 to 1500 CE. From there (between 1500 to early 1800), AfXoo slowly plateaued and gradually decreased. This period coincides with the introduction of O. sativa in West Africa in the early 16th century [5]. The AfXoo population then declined sharply (late 1800 to early 1900) and started to recover around the mid 20th century (Fig 3b). Estimated effective population sizes for O. glaberrima based on genomic data also show a decline after the 17th century [3] (Fig 3b).’

Overall, this paper has interesting and novel data, but makes claims beyond what the data shows. In addition, the manuscript suffered from various typos (i.e. ric instead of rice, hos instead of host) and a lack of copyediting throughout.

Response: Thank you for the suggestions, we have now modified the manuscript so that the claims reflect the data presented and we have corrected typos throughout.

Reviewer #2: This manuscript by Quibod et al presents a comprehensive and carefully executed population genomics analysis of African Xanthomonas oryzae pv. oryzae (AfXoo), integrating phylogenomics, recombination-aware analyses, demographic inference, and host domestication history. The dataset is extensive, the analytical framework is generally appropriate, and the evolutionary narrative is biologically compelling. My primary concern is overinterpretation of some evolutionary inferences, speculations, and sometimes framing it as causal relationship. Authors can revise their writing and tone down at some places. Overall, the study makes a valuable contribution to understanding pathogen diversification in the context of crop domestication and agroecosystem change.

Response: Thank you for your comments. We have revised the manuscript extensively to remove speculation specially in the results section and we have moved our hypotheses to the discussion. See details below.

Reviewer #3: The authors sequence a collection of 59 Xanthomonas oryzae pv. oryzae isolated from Oryza spp. in Africa (AfXoo) to assess their population structure, evolutionary history and diversity of transciption-activator-like effectors (TALEs). A tip-dated tree is shown and demographic inference is performed. TALE identification and repeat domain variation is assessed. Data from pathogenicity assays from previous studies is integrated to draw a link between TALE diversity and rice infection outcomes. Data from a previous study is also integrated to study TALEs diversity in field infections.

Response: Thank you for this assessment of our work.

# Part II – Major Issues: Key Experiments Required for Acceptance

Reviewer #1: The first issue is speculation about host specialization (AfXoo adaptation to native rice species vs. O. sativa). To empirically back these claims, there needs to be reciprocal inoculation experiments of AfXoo and AsXoo strains in various rice varieties. That would be a massive experiment, but would contribute substantially to the field. In the absence of this (which I doubt is feasible for this manuscript),

Response: This is a valuable suggestion but we believe it is beyond the scope of our paper, we are currently carrying out such experiments within the frame of a bigger project, which will still take some time to be finished, and given the scale of the experiments we believe they need to be in a separate publication.

I think the speculation about host specialization before and after the introduction of O. sativa should be scaled back, removed from all results, and limited to a short paragraph in the discussion. I would suggest a major reframe of the paper, focusing on allopatric pathogen evolution, and to contextualize the results in terms of comparison to the AsXoo population evolution.

Response: We have modified the manuscript to reduce the claims about host specialization in the result section. In the results section, as stated above, we still mention the date of introduction of *O. sativa* as context for comparison with the tip-dating and coalescent model results of AfXoo, but the hypothesis of the current populations being the result of the introduction is now in the discussion.

Our hypotheses on the impact of the introduction of *O. sativa* in the discussion read as follows:

PAGE 16-17, LINES 414-428.

‘This is suggested by our coalescent skyline plot model (Fig 3b) which shows two events of expansion of AfXoo interrupted by a bottleneck. We proposed that first, the emergence and proliferation of AfXoo in West Africa coincided with the rise of African cultivated rice (O. glaberima) 3,000 years ago [3]. Malian region is possibly the center of AfXoo diversification, O. glaberrima domestication happened in the inner Niger delta [3], and Mali harbors all the populations found; this could however be due to Mali being more heavily sampled. The introduction of Asian cultivated rice (O. sativa) in the early 16th century [5], and the subsequent decline of O. glaberrima [3], then gradually altered the population demography of AfXoo in the region, and served as a genetic bottleneck, such that the currently circulating populations are those capable of infecting both species. The AfXoo population has since overcome the resistance of O. sativa to varying degrees, with outbreaks of bacterial leaf blight in Africa in the late 1970’s and early 80’s [8,67–69]. Malian strains from currently circulating groups have been shown to indeed be able to equally infect both O. glaberrima (CG14) and O. sativa (cv. Azucena), with some strains even being more virulent in O. sativa, but not the opposite [48].’

Also as suggested, when discussing the differences between the Asian and African *Xoo* lineages we now state it within the context of allopatric speciation:

PAGE 15, LINES 373-381. ‘Allopatric speciation in bacteria has been shown as a result of geographical isolation [58] and in host-associated bacteria, the host can act as an additional isolation factor [59], such that the evolutionary history of some hosts can be traced by studying the pathogen [60]. The extent to which the geography or the adaptation to two hosts (*O. sativa* or *O. glaberrima*) can explain the differences between these two groups of *Xoo* is uncertain. In plant pathogens, domestication and monoculture have been proposed to favor allopatric divergence [61,62]. For *Xanthomonas axonopodis* for example, the existing host-specialized pathogenic pathovars have been proposed to emerge recently from broad host range ancestors, with their specialization (ecological isolation) driven by agricultural development [62]’

The second modification should be an effort to resolve the tree topology of lineages 1, 2, 3, and 4 with improved bootstrap support for critical nodes splitting these lineages. There should be phylogenetic support for a claim that BAPS lineages 1, 2, and 3 are “derived from” 4, or that claim should be removed.

Response: We have removed through the paper statements related to BAPS1 being derived from BAPS4 since indeed the topology of the tree seems to indicate another ancestry. We do state that BAPS, 2 and 3 shared an ancestor with BAPS4 since the topology of the tip-dated tree suggests this.

This is now stated in the discussion as PAGE 18, LINES 443-448:

‘Tip-dating analysis (Fig 3a) places the emergence of BAPS1-3 all after the introduction of *O. sativa*, while an ancestor of BAPS4 existed as early as 1000 CE (the timing of BAPS5 emergence could not be determined). We can imagine a scenario where the clonal populations emerge and expanded thanks to their ability to infect *O. sativa*, with BAPS2 and BAPS3 deriving from a BAPS4-like recombinant ancestor, while BAPS1 derived from an even more distant ancestor (Fig 3).’

We have also added bootstrap support to the tree shown in Figure 1, and posterior probability support for the tree in Figure 3, and state this more clearly in the legends.

Finally, there should be explicit tests for recombination in TALE and Xop sequences. I would suggest looking at Gubbins, FastGear, or ClonalFrameML to explicitly check for recombination.

We did whole genome scans for recombination using Gubbins, and we then mapped the SNPs within the recombination tracts to corresponding TALE or Xop loci using the BAI3 strain.

These were not possible to do explicitly for TALEs due to their repetitive nature, but we were able to perform a comparison of pairwise distances between TALEs and phylogenetic distance between strains, identifying TALEs that have diverged significantly between closely related strains. The changes in these TALEs involved multiple repeats and could be the result of different processes, including recombination,. We state these in the manuscript as rearrangements.

Reviewer #2: (No Response)

Reviewer #3: This will be an important resource of general utility and specialist interest, but some changes in data presentation, writing and integration of prior work will strength the quality of the manuscript:

1. The integration of work from previous studies was either patchy (pathogenicity results) or poorly connected to the main results (Diallo et al Bagre field populations). Although both sections ask interesting questions, the prior data is either insufficient (pathogenicity data) or the findings are not sufficiently well placed within the context of the other findings shown here. The Bagre field collection is not described and it is unclear how the results in Fig. 4 are linked with data in Fig. 1-3. This section can be improved with clearer writing and by adding additional detail to relevant figures (e.g. highlighting the 18 Bagre strains in Fig. 1).

Response: We have changed the way these sections are presented to integrate them better with the rest of the paper and make them clearer for the reader.

The Bagré data we now present mostly in the context of identifying changes in TALEs with strains collected in a short time span and in a small geographical region. We pinpoint the timing of the common ancestor for these strains and we highlight that the diversity in TALEs originated in a short time from rearrangements. We state PAGE 12-13, LINES 300-317:

‘We were also interested in examining the variation of TALEs in the field, in a relatively small geographic area and in a short span of time, by reexamining TALE variation data from fields in the Bagré region of Burkina Faso [35]. In our previous study we found in a collection of 177 strains collected in Burkina Faso from 2003 to 2018, eight distinct TALome patterns as shown by Restriction Fragment Length Polymorphism (RFLP). To understand the genomic changes behind this variation, we sequenced 15 of these strains collected in nine fields in Bagré between 2016 and 2018 which were regularly prospected; the genome sequences showed TALE content that correlated with the RFLP patterns (S13 Fig).

All the sequenced strains from Bagré belonged to BAPS1 and are estimated to have a common ancestor that existed around as early as the 1930s (Figure 3a), so TALE variation found in this group can be considered recent. As expected from the RFLP, we found rearrangements in TALE sequences, between these closely related strains (Fig 5b). These changes include terminal reassortment [56] between TalA and TALI to create a new T3E in strain CIX4067 (Fig 5c and d), deletions of two repeats in TalA in strain CIX3296, tandem duplication of the complete TalB sequence in strains CIX3025 and CIX4114, complete deletion of TalI in strain CIX4033, and pseudogenization of TalE due to a frameshift near the Type III secretion signal in five of the strains (S14-S15 Fig). This highlights how TALE sequence diversity can arise in a short time potentially creating phenotypic diversity.’

We have modified Figure 1a to highlight the Bagré strains (blue bold font) in the tree. We have also modified Figure 5 to show now more clearly the different TALE alleles found in all the Bagré strains and highlight which strains were collected in the same field and in which years. We have also modified Supplementary Figure 13b, to show better how the genomic data compares to the previously shown RFLP.

Regarding the comparison with the pathogenicity results we are unfortunately limited by what is available but we have made efforts to make this section clearer to the reader. We now mainly focus on correlating when possible effector content with reported virulence profiles, as in the case of strains being controlled by Xa1 because they do not possess iTALEs, and we highlight the intriguing case of strains are controlled by Xa7 despite not carrying a matching TALE.

We have also added a Supplementary dataset (S5 Dataset) detailing all the available pathogenicity data for AfXoo.

2. It is reasonable to hypothesize there is a connection between the arrival of a new Oryza species and the diversification of AfXoo, but I do not see how the coalescent skyline plot and tree provide clear and undisputed evidence of a strong bottleneck in AfXoo corresponding to O. sativa introduction. Some recent clonal expansions were sampled, but a diverse recombinant population is also still circulating. The fact isolates are recovered from other Oryza species - including a wild species - suggests the situation is more complex.

Response: We have now removed the claims of causality between the introduction of *O. sativa* and the bottleneck from the result section. The date of introduction is mentioned in the results for context, and the hypotheses of an impact of *O. sativa* is fleshed out in the discussion as follows PAGE 16-17, LINES 414-428 :

‘This is suggested by our coalescent skyline plot model (Fig 3b) which shows two events of expansion of AfXoo interrupted by a bottleneck. We proposed that first, the emergence and proliferation of AfXoo in West Africa coincided with the rise of African cultivated rice (O. glaberima) 3,000 years ago [3]. Malian region is possibly the center of AfXoo diversification, O. glaberrima domestication happened in the inner Niger delta [3], and Mali harbors all the populations found; this could however be due to Mali being more heavily sampled. The introduction of Asian cultivated rice (O. sativa) in the early 16th century [5], and the subsequent decline of O. glaberrima [3], then gradually altered the population demography of AfXoo in the region, and served as a genetic bottleneck, such that the currently circulating populations are those capable of infecting both species. The AfXoo population has since overcome the resistance of O. sativa to varying degrees, with outbreaks of bacterial leaf blight in Africa in the late 1970’s and early 80’s [8,67–69]. Malian strains from currently circulating groups have been shown to indeed be able to equally infect both O. glaberrima (CG14) and O. sativa (cv. Azucena), with some strains even being more virulent in O. sativa, but not the opposite [48].’

We further support this hypothesis now by citing other cases where plant pathogen adaptation to a new host leads to a similar population structure. PAGE 17, LINES 429-440:

‘While it is possible that other factors explain the current and past demography of AfXoo (e.g. environmental changes), the two-step adaptation to two domesticated hosts is a compelling hypothesis. Similar population structures involving bottlenecks and clonal expansion have been reported in other pathogens to be associated with host adaptation [41,70], and loss of genetic diversity might be a feature of a pathogen adapting to domesticated crops [38]. Phylogenomic analyses of North American isolates *Erwinia tracheiphila*, causal agent of bacterial wilt in cucurbits, showed the population is composed of expanding clusters with low genetic heterogeneity that might have emerged to infect cultivated cucumber Cucumis spp. after its introduction to the Americas in the early 1500 [71]. And similarly, emerging outbreaks of *Pseudomonas syringae* pv. *actinidae* infecting kiwifruit are being caused by genetically homogeneous lineages arising from a source population in a process that might be related to the recent domestication (20th century) of this host [72].’

Finally, we now explicitly acknowledge in the discussion that indeed the situation of AfXoo might be more complex and that there is likely diversity that has not been sampled. PAGE 18, LINES 445-454:

‘The clonal populations might have emerged and expanded thanks to their ability to infect O. sativa, with BAPS2 and BAPS3 deriving from a BAPS4-like recombinant ancestor, while BAPS1 derived from an even more distant ancestor (Fig 3). This said, our dataset and analyses are biased precisely because sampling has mostly been made in cultivated, symptomatic O. sativa (because of its economical importance) with few samples coming from O. glaberrima or wild rices. The full diversity of AfXoo is likely yet to be explored and it might encompass populations not associated with cultivated rice fields. The fact that the diverse BAPS4 and the genetically distant strain MAI134 (BAPS5) were found circulating relatively recently, might hint there is a hidden diversity of AfXoo to be sampled.’

3. Experimental data showing differences in pathogenicity due to variation in TALE alleles would be highly valuable of course.

Response: As in the case of reciprocal inoculation experiments suggested by reviewer 1, we believe this data, while desirable, is beyond the scope of our paper. We are indeed in the process of studying how different AfXoo TALEs induce genes in different rice species and their reaction to pathogenicity, but these analyses are large enough (and still unfinished) to be the subject of a separate publication.

# **Part III – Minor Issues: Editorial and Data Presentation Modifications**

Reviewer #1: General notes
The code is not publicly available - the link is not accessible.

Response: The code is now accessible at https://forge.ird.fr/phim/quibod/african_xoo

The citations were clearly from Zotero, but still linked. Unlink citations from Zotero for submissions, and also the font / size of the citations was different from the surrounding text.

Response: The citations are unlinked in the final document.

The figure legends dropped in the middle of the text was confusing.

Response: We were following the Plos Pathogens template, we now moved the Figure legends to the end of the manuscript.

Figures
Figure 1: It says in the legend that it was constructed with 1,000 bootstrap, but there are no labels with bootstrap support for individual nodes. What was the outgroup for this? The white boxes for host (presumably “unknown”?) are not defined. In B, the pie charts are cut off. The country names are included for East Africa but not West Africa

Response: We now indicate in Figure 1 bootstrap values >80% as gray dots in node. We indicate in the Figure legend that strain MAI134 was chosen as an outgroup. We define the ‘unknown’ category. And we make sure in b) both pie charts and country names are not cut-off.

Figure 3: This the phylogenetic tree that makes me suspicious of the claim that lineages 1, 2 and 3 are derived from BAPS 4.

Response: Indeed the topology here seems to point to BAPS1 sharing a more distant ancestor with BAPs4 than the other populations, our hypothesis was mainly derived from the shared alleles and reticulated tree in Figure 2. We have now modified the text and we do not mention BAPs1 being derived from BAPS4.

Figure 4A has no x-axis label.

Response: We have added the title of the x-axis as “RVD position”

Figure S1 - can’t read text labels for Mali and Niger. Also clarify in the figure legend that the 333 strains were only included for the VNTR testing, while 59 were using in WGS.

Response: We have made the country labels with a white background for readability, and we now state in the legend that these strains were used for VNTR analyses and a subset was used for whole genome sequencing.

Figure S2 - use the same colors for each country that were used in Fig. 1 and Fig. S1.

Response: We now use the same country colors in Figure S2 as in all other Figures in the paper (Fig1, Fig3, Fig S1)

Figure S3 - There are discrepancies in groupings between DAPC and BAPS that are not addressed anywhere.

Response: These discrepancies are mainly due to the distance thresholds used to define groups when using MLVA DAPC as well as factors like recombination and polyphyly, we now state this in the results

PAGE 7, LINES 170-173: ‘The groups defined by BAPS also largely agree with clustering based on previous [11] and our current MLVA result (S2 Fig), but discrepancies arise due to different methods using different genetic distance thresholds to establish groups as well as confounding factors like the polyphyly of BAPS4.

Figure S5 - BAPS-5 is included here but not above in S4 (i.e. strain Mai134). Why?

Response: We had initially removed it from S4 to better visualize the separation between BAPS1-4 since we had already shown it to be a distant group. We agree this might cause confusion so we now include BAPS5 in both S4 and S5.

Figure S7 - The ‘highlight’ for strain Mai134 that is mentioned in the figure legend is missing. For C, there are three nodes with dates, but it is not clear why these nodes were selected?

Response: This is now Figure S8, the highlight was a yellow dot that indeed was not easily visible, we now add the name of the strain in the figure. The selected nodes represent the MRCA for BAPS1, 2, and 3, as well as the whole tree; this is now indicated in the legend.

Figure S10 - how was this recombination inferred? The presence of a shared sequences does not necessarily prove recombination.

Response: That is correct, here we were identifying shared blocks of repeats that could potentially arise from homologous recombination / gene conversion, but we were unable to test this.

We now refer to these blocks in the results as PAGE 11, LINES 281-285: ‘However, we also detected in the RVD alignments changes involving several contiguous repeats, these include indels from 1 to 7 repeats (i.e. alleles 4-6 in TalB) tandem repeat duplications (i.e. allele TalH2) (Fig4a) as well as blocks of shared repeats between different TALE loci which might have evolved to bind to similar DNA motifs or could be the result of gene conversion events (S11 Fig).’

And the Supplementary Figure Legend states: **Figure S11. Shared blocks of repeats were identified between TalF and other TALEs loci.** The graph shows blocks of repeats shared between different loci, suggesting rearrangements, potentially resulting from gene conversion events in some parts of the TALE repeat region as highlighted in the red block.

Results
The logic that lineages 1, 2, and 3 have lower r/m and therefore may be derived from lineage 4 (with higher r/m) does not follow. It is possible that higher r/m in lineage 4 is from introgression from lineages 1, 2, or 3, hence has greater diversity - not that it has greater diversity because it is the ancestral lineage.

Response: We have removed suggestions that lineage 4 is the ancestral lineage from this section, we now state that it is more recombining and diverse than the other populations. We have also added results from new recombination analyses as stated above, showing that it acts both as a donor and recipient of recombinant alleles (Figure S6b) compared to the other population.

Lines 280-289 - only present what your actual data shows. That is, the inferred Xoo population sizes over time. All speculation about the influence of rice species should be moved to the discussion.

Response: We modified this section to describe the coalescent model. We keep the date of introduction of *O. sativa* for context, and we added information on the estimated effective population size for *O. glaberrima* from Cubry et al. (2018) during this same period.

Lines 324- 329 - mostly an explanation of background and other studies, should be moved to introduction.

Response: We moved this paragraph to the result section relating to phenotype and TALE content, we reduced the background and now focus on the results stating that we did not find any iTALEs in our genomes and that as consequence most AfXoo strains were controlled by Xa1 in the available phenotypic data.

Lines 337 - 338 - no explanation of how recombination was identified.

Response: We rephrased this to state that we identified blocks of shared repeats
Lines 370-374 speculation

Response: We deleted this.

Lines 396-398 - IR24 and IRBB are not defined - please explain for non-rice specialists.

Response: We have added a sentence explaining the nomenclature, now reading

PAGE 13, LINES 321-328: ‘To investigate the virulence capability of AfXoo we compiled phenotype data (S3b Fig; S5 Dataset) from various sources that used near-isogenic lines (NILs) containing single resistance genes, mainly sourced from O. sativa cultivars [10,12,36–38]. These NILs, (named IRBB1, IRBB3, …) carry known resistance genes against Xanthomonas (named Xa1, Xa3, respectively) in the genetic background of IR24, a cultivar normally susceptible to AsXoo strains [50]. We observe that populations BAPS3-5 were unable to infect IR24, and accordingly could not infect any IRBB lines (S3b fig). IR24 may carry a resistance gene able to control these AfXoo populations [51].’

We also now point the readers here and in the methods to a review paper (Lore et al., 2025) describing these lines in detail.

Lines 403-405, 408-410 - not results, should be moved to introduction or discussion

Response: we have removed these sentences

Discussion
Line 436 - missing parentheses after “ago” i.e. (9,000 years ago)

Response: we have added this parenthesis

Line 437 - The evolution of a highly aggressive phenotype is not a predetermined outcome. There is selection for higher fitness, but not always higher aggressiveness.

Response: we have removed this sentence

Line 438 - “In africa” the “In” is capitalized incorrectly

Response: we have corrected this

Line 435-445 remove the word “evidence” - this is a suggestion for this hypothesis, not evidence

Response: We modified this sentence to state PAGE 16, LINES 414: ‘This is suggested by our coalescent skyline plot model’

Line 461-465 - “We can imagine a scenario… “ speculation

Response: We removed this to now read PAGE 18, LINES 445-446 : ‘The clonal populations might have emerged and expanded thanks to their ability to infect *O. sativa*’

Line 482 - “ric” instead of “rice”

Response: We have removed this sentence

Line 521 “While the changes..” this is not a complete sentence.

Response: We have removed this paragraph.

Line 526 “Here, we demonstrate…” remove “demonstrate” and replace with “hypothesize”

Response: We have removed this paragraph. The current version states more simply PAGE 19, LINES 479-480: ‘our results hint at the influence of crop domestication in the emergence and spread of pathogens, as reported in other systems [44,80].’

Line 528 - “adapted” - was not actually shown

Response: We have removed this sentence.

Methods
There were two different methods using for core genome SNP extraction. The first (for the entire Xo species) was using panaroo and snp-sites, and then after for Afxoo, the method changed to using parsnip and gubbins. This is not justified, and it is confusing to use two distinct methods.

Response: We used Panaroo and snp-sites for extracting SNPs in the *Xo* species because with Panaroo we can capture more of the alleles which parsnp can’t since it applies whole genome alignment. In addition, we weren’t interested in the recombination between phylogroup/pathovars that is why we didn’t apply Gubbins after. For AfXoo, we used parsnp because these strains are closely related and it takes into account alignment in the intergenic regions which Panaroo couldn’t. Also, we can create a reference based vcf file with parsnp that we need when we mapped the recombinant SNPs. We add the justification in the method section PAGE 22-23, LINES 564-578.

To build a multiple core gene alignment, genes were first clustered into orthologous groups using Panaroo v1.2.10 [89] in strict mode, paralog clusters were merged, and invalid annotated genes were removed. SNPs were extracted from the core gene alignment using snp-sites v2.5.1 [90], and used to create a ML phylogeny by IQtree v2.2.2.6 [91]. This SNP extraction method was performed since Xanthomonas oryzae phylogroups are highly divergent, and the pangenome captures more of the diversity. Parameters for phylogeny construction were set with the ultrafast bootstrap at 1000, specifying the substitution model GTR+I+Γ, and the Xanthomonas oryzae strain from the US, X11-5A, as the outgroup. To estimate accurate branch lengths, invariant site information was added, and was obtained from snp-sites.

The phylogeny for AfXoo was carried out by first conducting a multiple alignment to acquire the core genome and extract single nucleotide polymorphism (SNP) with BAI3 as the reference from the core alignment using parsnp v1.5.6 which is included in the Harvest suite tools [92]. This SNP extraction method is mostly used for closely related strains and it also captures the intergenic regions which Panaroo cannot.

Line 611 - which SNP alignment was used for Splits tree?
In general, clarify when the core genome SNP matrix was used with recombination or without recombination.

Response: For SplitsTree, we used the SNP matrix with recombination, this is now stated in the methods and in the Figure legend.

Line 640 - 660 - the inclusion of Mai134 or not is confusing throughout this. There are either four or five k / populations, but it is unclear why Mai134 was included or not in each of these analyses.

Response: To avoid confusion, in the revised version we include MAI134 in the majority of analyses. The exceptions are the genetic diversity analyses shown in Figure 2, and Figure S6b, where this strain could not be included as these analyses require a representative number of samples. We state this in the corresponding Figure legends and the result section as PAGE 8, LINES 189-190: ‘Because it is represented by a single strain, BAPS5 was excluded from analyses sensitive to sample size.’

We also exclude BAPS5 from the tip -dating analyses after showing the root-to-tip regression is not possible when including this strains (Figure S8a)

Line 658 - Fst - pairwise?

Response: Modified to state pairwise fixation index (Fst)

Lines 674-677 - missing details on which rice genes?

Response: We have added the names and IDs of the genes here (PAGE 26, LINES 657-658), and they can also be found in Supplementary Table 1.

‘Only the known TALE-rice targets with a role in susceptibility were shown: *SWEET14*, *TFX1*, and *ERF#123* [[15,57]](https://www.zotero.org/google-docs/?VqkHk5) (S1 Table).’

Reviewer #2: Below are my comments/suggestions.

Abstract: line 82-84. Is it evolutionary force acting upon rice? Or other way around?

Response: We have modified the author's Summary to make it less technical, this sentence was deleted.

Fig S1 could benefit from having years of isolation indicated alongside country and number of strains. It is great that authors have used different figures to explain the vast dataset, but sometimes, looking at the map and knowing the timespan in which strain collection is done is helpful.

Response: We have added a bar plot at the top right corner of the graph to show the temporal data of the isolates.

Fig 3a: Yellow points- are those AfXoo strains clustering with Asian Xoo? I assume they are not in subsequent analyses? If included, would there be shared alleles among those strains and any AfXoo lineages?

Response: In Figure S3a, these are recent outbreaks of Asian *Xoo* resulting from two independent introductions in East Africa and Madagascar. These strains are described in Schepler-Luu et al. 2023 (Tanzania outbreak) and Rabekijana et al., 2026 (Madagascar outbreak). They are, for our purposes, Asian strains and the genetic differences between AfXoo and AsXoo stated in the paper hold true for these strains. To our knowledge they do not share more alleles with AfXoo than other AsXoo strains.

My primary concerns are overinterpretation of some evolutionary inferences, speculations, and sometimes framing it as causal relationship. Reframing these or perhaps, stating the alternative explanations would be beneficial for the readers. Below are some of these examples:

Response: We have throughout the paper, specially in the results section, reduce speculation and leave our hypotheses for the discussion.

1. Line 246-247. How is this evolutionary trajectory inferred about adapting different host resistances? Is this based on phenotypic data?

Response:This sentence has been deleted

2. Inferred ages on the tip-dating analysis primarily reflect allele histories rather than lineage emergence per se.

Response: We have added a precision stating this as PAGE 9-10, LINES 238-240: ‘We used phylogenetic molecular clock reconstruction to estimate when the most recent common ancestor (MRCA) of AfXoo groups existed according to allele histories.’

3. BAPS4 is polyphyletic, and shares alleles with other BAPS. This lineage is ancestral-like recombining reservoir from which multiple monophyletic, clonal lineages emerged and this is supported by the data presented by the authors. This is a reasonable and supported explanation. However, authors need to be cautious about using a term founder population.

Response: We have removed through the paper claims that BAPS4 is the founder population, we instead now highlight that is more recombining than the other BAPS and that it shares alleles with them.

PAGE 9, LINES 223-226: ‘Overall, these results indicate that BAPS1-3 are clonal populations differentiated from each other and possibly originating from relative recent bottlenecks, while BAPS4 is a recombinant population that shares alleles with the clonal populations as well as with the more genetically distant BAPS5.’

And we suggest in the discussion, that the clonal populations could derive from a population similar to BAPS4.

PAGE 18, LINES 445-447: ‘The clonal populations might have emerged and expanded thanks to their ability to infect *O. sativa*, with BAPS2 and BAPS3 deriving from a BAPS4-like recombinant ancestor’

Observation that BAPS4 strains exhibit no virulence in any of the tested lines, despite persisting in the populations and being largely associated with O. sativa (only 1 strain from other Oryza)- this is intriguing and warrants a brief discussion. Even if ancestral-like, the long-term persistence of these avirulent (?) strains can mean some interesting ecological fitness factors? Were these strains pathogenic on the host of isolation?

Response: BAPS3, 4 and 5 did not exhibit virulence in the tested line likely because they are all derived from the same background, IR24. The strains were collected from symptomatic leaves so they are likely able to infect other backgrounds. Data for other rice varieties other than IR24 is not available for as many strains but we have now added phenotyping data from two *O. sativa* accession (Azucena and Komboka) and one *O. glaberrima* (CG14) to S5 Dataset, which show that strains from these groups can be symptomatic in other backgrounds.

We do also agree that the persistence of this more diverse group is intriguing and we now state in the discussion.

PAGE 18, LINES 448-454: ‘This said, our dataset and analyses are biased precisely because sampling has mostly been made in cultivated, symptomatic *O. sativa* (because of its economical importance) with few samples coming from *O. glaberrima* or wild rices. The full diversity of AfXoo is likely yet to be explored and it might encompass populations not associated with cultivated rice fields. The fact that the diverse BAPS4 and the genetically distant strain MAI134 (BAPS5) were found circulating relatively recently, might hint there is a hidden diversity of AfXoo to be sampled.’

4. Another statement: introduction of O. sativa into Africa served as a bottleneck in AfXoo population is another place where narrative is somewhat appearing as causal. While demographic contractions inferred are consistent with domestication of two rice species, coalescent based effective population size estimates alone cannot establish the causation. Alternative explanations such as sampling bias, or changes in agricultural practices other than host, or climate or combination thereof, remain plausible. So more cautious formulation might be useful here to show biological interpretation by the authors.

Response: We have modified the document to reduce claims of causality between the *O. sativa* introduction and the bottleneck, specially in the results section.

The description of the bottleneck now reads in the results as PAGE 10, LINES 249-256: ‘Next, we examined the population dynamics of AfXoo over time according to the coalescent model (Fig 3b). The model initially showed a gradual increase in effective population size between 500 to 1500 CE. From there (between 1500 to early 1800), AfXoo slowly plateaued and gradually decreased. This period coincides with the introduction of *O. sativa* in West Africa in the early 16th century [5]. The AfXoo population then declined sharply (late 1800 to early 1900) and started to recover around the mid 20th century (Fig3b). Estimated effective population sizes of *O. glaberrima* based on genomic data also show a decline after the 17th century [3] (Fig3b).’

We now acknowledge alternative explanations, and state that host adaptation is a hypothesis PAGE 17, LINES 429-433:

‘While it is possible that other factors explain the current and past demography of AfXoo (e.g. environmental changes), the two-step adaptation to two domesticated hosts is a compelling hypothesis. Similar population structures involving bottlenecks and clonal expansion have been reported in other pathogens to be associated with host adaptation [41,70], and loss of genetic diversity might be a feature of a pathogen adapting to domesticated crops [38]’

5. TalF variants in BAPS1 lacking virulence. Is this demonstrated only in one strain or multiple? How are authors coming to the inference that TalF has no role in virulence?

Response: This has been shown so far for only one strain, BAI3. We now state PAGE 14, LINES 351-353:

‘The TalF allele of the strain BAI3 (BAPS1) was shown to be unable to induce OsSWEET14 and did not contribute to virulence in plants [15]. It is unknown whether other TalF alleles have virulence activity.’

6. Mali as center for diversification: Isn’t Mali heavily sampled here? How can it be distinguished from true evolutionary origin?

Response: We have added this caveat.

PAGE 17, LINES 417-420: ‘The Malian region is possibly the center of AfXoo diversification, O. glaberrima domestication happened in the inner Niger delta [3], and Mali harbors all the populations found; this could however be due to Mali being more heavily sampled.’

Reviewer #3: Clarity of text from L117 onwards can be improved, there are abundant sentence fragments and strange sentence constructions. Some examples:

L118 - sentence begins with 'And'

Response: Modified as. PAGE 4, LINES 99-102: ‘*Xoo* T3Es can be classified in two groups: Xanthomonas outer proteins (Xop) involved in suppressing immunity [19], and Transcription Activator-Like Effectors (TALEs) involved in inducing physiological changes in the host that increase susceptibility [20]’

L128 - 'TALE diversification ... has been shown to play an active role in shaping the diversity of TALEs'. Circular.

Response: Modified as: PAGE 4-5, LINES 111-112: ‘TALEs display high diversity in sequence and function, linked to their propensity for recombination, insertion and deletions in the repeat region’ [29].

L172 - 'These strains formed a single node'. Meaning they are monophyletic?

Response: Modified as: PAGE 6, LINES 161-162: ‘In total, 87 AfXoo genomes were analyzed, these strains were monophyletic in a *Xanthomonas oryzae* core gene phylogeny (S3a Fig).’

L334 - 'The RVD alignment also showed indels in some TALEs, and could go as long as seven to two gaps'. Improve phrasing.

Response: Modified as: PAGE 11, LINES 279-285: ‘Several point mutations were observed in the TALE families, ranging from nine (TalF) to a single (TalC) RVD amino acid change. However, we also detected in the RVD alignments changes involving several contiguous repeats, these include indels from 1 to 7 repeats (i.e. alleles 4-6 in TalB) tandem repeat duplications (i.e. allele TalH2) (Fig4a) as well as blocks of shared repeats between different TALE loci which might have evolved to bind to similar DNA motifs or could be the result of gene conversion events (S11 Fig).’

L396 - 'BAPS3 and 4, were unable to infect IR24, an AsXoo susceptibility check, as this particular rice background was shown to contain the Xa18 resistance gene'. Improve phrasing.

Response: Modified as: PAGE 13, LINES 326-328: We observe that populations BAPS3-5 were unable to infect IR24, and accordingly could not infect any IRBB lines. IR24 may carry a resistance gene able to control these AfXoo populations [[51]](https://www.zotero.org/google-docs/?broken=yuFLlH).

L432 - 'fastly spreading'

Response: this line was deleted

L438 - 'Meanwhile, In Africa, domestication of O. glaberrima was relatively recent (3,000 years ago), and this is likely reflected in its genetic diversity, with O. glaberrima having less genetic variation compared to O. sativa'. Improve phrasing.

Response: this line was deleted

L628 - 'The detailed parameters for TreeTime are resolving polytomies, calculation of confidence intervals by marginal posterior distribution, and maximal iteration of 1000 for maximum optimization.' Those are not detailed parameters.

Response: Modified as: PAGE 24, LINES 605-608:

‘The detailed parameters for TreeTime are as follows: --stochastic-resolve (resolving polytomies), --coalescent skyline (estimate changes in the population) --gtr infer (GTR model) --confidence (divergence time confidence interval) and --max-iter 1000 (optimizing the best tree search).’

***

175 - BAPS4 is very interesting. The cluster includes recombinant genomes and is polyphyletic in the tree. The differences for BAPS4 in diversity and Tajima's D are unsurprising given these are recombinant genomes.

Response: We agree, we have modified the section related to genetic measures to emphasize the recombinant nature of BAPS4.

L185 - No support values are shown on the tree in Fig. 1. Some unresolved relationships and lack of support evident in Fig. S3. Figure display for S3 can be improved.

Response: We have added bootstrap support to Fig 1a and Fig S3.

L197 what is meant by BAPS2 emergence coinciding with loss of BAPS3? Were BAPS2 and BAPS3 isolated from the same precise location in Mali? BAPS2 looks like a recent clonal expansion in Fig.1, if these were all sampled from one location in Mali it is likely BAPS2 recently spread in that area.

Response: This was deleted, there might not be enough sampling to assess that BAPS3 has been lost.

L229 - Recent expansion is very likely given Fig. 1, and recombination is more likely than balancing selection given the data shown. Strange dashed scale bars.

Response: We agree but we think it is adequate to list alternative possible explanations. We have changed the dashed scale to solid.

L239 - BAPS1-3 have recovered more rapidly from what?

Response: This line was deleted

L246 - It is speculative to conclude the presence of unique alleles in a population is due to their adaptation to different host genetic resistances or niches, this could be drift.

Response: This line was deleted.

L264 - 'reconstruction revealed that the emergence of the AfXoo group occurred around the year 656 CE (common era) (334 BCE-1351 CE 95%HPD) during the period of intensive cultivation of African rice O. glaberrima'. The period referred to here is more appropriately the entire 1000 year interval predating O. sativa introduction.

Response: Modified as: PAGE 10, LINES 240-242: ‘Our analyses suggests that the MRCA of all the AfXoo studied existed around the year 656 CE (common era) (334 BCE-1351 CE 95%HPD) when *O. glaberrima* was the only cultivated rice in Africa’

L280 - Rather than a recent bottleneck on O. sativa, is it possible the apparent reduction is due to having sampled 3 recently expanded clonal lineages that share distant ancestry with each other? Coalescent inference of past demography (along with tip dating) produces statistical ranges based on underlying assumptions about the nature of the data.

Response: We now acknowledge that our dataset might be biased and that the diversity of AfXoo might be greater than what has been sampled. We state PAGE 18, LINES 447-454: ‘This said, our dataset and analyses are biased precisely because sampling has mostly been made in cultivated, symptomatic *O. sativa* (because of its economical importance) with few samples coming from *O. glaberrima* or wild rices. The full diversity of AfXoo is likely yet to be explored and it might encompass populations not associated with cultivated rice fields. The fact that the diverse BAPS4 and the genetically distant strain MAI134 (BAPS5) were found circulating relatively recently, might hint there is a hidden diversity of AfXoo to be sampled.’

L311 - 'a single BAPS4 isolate that contains exactly the same TALE family allele with some BAPS1 strains' - this is interesting, worth pursuing experimentally.

Response: We re-examined the raw reads for this isolate and we believe there might have been a mix of strains sequenced together by accident, we have excluded this genome from all analyses in this new version.

L314 / Fig. 4a - Are there alternative methods of visually summarising TALE diversity? An alternative to a colored table must be possible

Response: Displaying the RVD sequences with colors to highlight the diversity we believe to be ideal and informative, and in this figure it allows us to relate the alleles with their frequency. This representation has previously been used in other publications dealing with AfXoo TALEs (Tran et al., 2018; Doucouré et al., 2018) and we opt to keep it for consistency.

L323 - whole paragraph of speculation about the absence of iTALES in the African Xfoo pathogen population and their presence in a different Asian population, which is not the subject of this paper. Move to Discussion.

Response: We moved this to the section related to the phenotype data, and now specifically relate the lack of iTALs with the reported phenotypes in line IRBB1.

L343 - Here the paper takes a strange turn. Authors explain they examined the spatiotemporal resolution of TALE effectors in the field, by 'reexamining data' from another paper. It is not clear what connection the data in that paper has to the work shown up to this point. Where is Bagre relative to the sample areas in Fig. 1?

Response: We extensively modified this section to make it more understandable. We highlight that these strains were sequenced to assess TALe variation in a short time span and in a short geographical area.

This section now states:

PAGE 12, LINES 300-317: ‘We were also interested in examining the variation of TALEs in the field, in a relatively small geographic area and in a short span of time, by reexamining TALE variation data from fields in the Bagré region of Burkina Faso [36]. In our previous study we found in a collection of 177 strains collected in Burkina Faso from 2003 to 2018, eight distinct TALome patterns as shown by Restriction Fragment Length Polymorphism (RFLP). To understand the genomic changes behind this variation, we sequenced 15 of these strains collected in nine fields in Bagré between 2016 and 2018 which were regularly prospected; the genome sequences showed TALE content that correlated with the RFLP patterns (S13 Fig).

All the sequenced strains from Bagré belonged to BAPS1 and are estimated to have a common ancestor that existed around as early as the 1930s (Figure 3a), so TALE variation found in this group can be considered recent. As expected from the RFLP, we found rearrangements in TALE sequences, between these closely related strains (Fig 5b). These changes include terminal reassortment [56] between TalA and TALI to create a new T3E in strain CIX4067 (Fig 5c and d), deletions of two repeats in TalA in strain CIX3296, tandem duplication of the complete TalB sequence in strains CIX3025 and CIX4114, complete deletion of TalI in strain CIX4033, and pseudogenization of TalE due to a frameshift near the Type III secretion signal in five of the strains (S14-S15 Fig ). This highlights how TALE sequence diversity can arise in a short time potentially creating phenotypic diversity.’

L349 - New strains were sequenced from Bagre?

Response: We sequenced strains that were previously typed by RFLP.

L355 - Were all 18 Bagre strains already shown in Fig. 1? Which are these exactly among the 30 Burkina Faso genomes? Membership in BAPS cluster 1 is mentioned but the relatedness or identity between the 18 Bagre isolates from Diallo et al. to the 59 sequenced for Fig. 1-4 is not clearly explained. I understand the idea in principle, but often struggled to connect the data from Diallo et al. / Fig. 4 to Fig. 1-3.

Response: We now indicate the Bagré strains in Figure 1, names of the strains are colored in blue. We also show the node corresponding to the MRCs of Bagré strains in Figure 3. And we mention these strains early in the manuscript.

When describing our genome dataset in the result section we state PAGE 6, LINES 154-160:

‘To have a representative genome set for AfXoo, based on the MLVA results, we selected 56 strains with different haplotypes, geographical origins and/or hosts of isolation for whole-genome long-read sequencing. To also assess diversity at a small geographical scale, a subset of our sequences corresponded to 15 strains isolated in the Bagré region in Burkina Faso, which had been previously shown, based on Southern Blots, to contain variable TALE repertoires [35]. We combined our dataset with other available complete genome sequences from NCBI (S2 Dataset).’

Which of the (e.g.) six TalA alleles in Fig. 4a correspond to the two TalA alleles in Fig. 5a? How is the TALome pattern in Fig. 5a linked with the TALE alleles in Fig. 5a? Why does CIX3092 have TALome pattern 2 and not 5?

Response: We have modified Figure 5 to reduce confusion. We do not mention the ‘TALome patterns’, we keep the correspondence between the RFLP and the genomic data for SFig 13. Instead we show the TALE content for all the Bagre strains, the TALE alleles are shown as colored numbers and these numbers correspond to the numbers in Figure 4a. This is indicated in the legend.

TALE allele presence for 8 genomes is shown in Fig. 5a. Since these strains appeared the tree in Fig. 1, is the data for the 10 additional strains shown only in the Fig. 5b barchart? There are 30 Burkina Faso genomes in Fig. 1 - are the 18 Bagre isolates a subset? A partially different set? Statements like 'Since the strains analyzed share the same node (S3b Fig),' are difficult to interpret: presumably this means the BAPS1 isolates are monophyletic, but which node? There are several, 'same node' could mean very different things across the tree.

Response: We now show all the Bagré strains alleles in Figure 5. We clarify that they are a subset and highlight them in Figure 1. And we indicate their node in Figure 3.

L393 - The last section also compiled some pathogenicity assay results from multiple studies to draw conclusions about how TALome influences infection in the field. This is more easily integrated in this paper but given the heterogeneous sources of this data and patchy TALome representation, I think it is mainly useful for hypothesis generation.

Response: We agree, we have reduced this section to state results that explicitly make connections between TALE content and reported phenotypes, and move some parts to the discussion.

L398 - What are IRBB lines? What are xa5 and xa7?

Response: We clarify this now in the result section PAGE 13, LINES 321-326: ‘To investigate the virulence capability of AfXoo we compiled phenotype data (S3b Fig; S5 Dataset) from various sources that used near-isogenic lines (NILs) containing single resistance genes, mainly sourced from O. sativa cultivars [10,12,36–38]. These NILs, (named IRBB1, IRBB3, …) carry known resistance genes against Xanthomonas (named Xa1, Xa3, respectively) in the genetic background of IR24, a cultivar normally susceptible to AsXoo strains [50].’

Methods L547 - the summary of integration of pathogenicity assay from other studies should be in a different Methods section.

Response: This is now in a separate ‘Collecting the phenotypic data’ section.
