# Supplementary material for: Two domesticated species of rice shaped the population structure of Xanthomonas oryzae pv. oryzae in Africa
Source: PLoS Pathog. 2026 Sep 17;22(9):e1014015. doi: 10.1371/journal.ppat.1014015 (PMC13619019; doi:10.1371/journal.ppat.1014015)
Supplement: Peer review history

# Peer review: Decision Letter 0

**DOI:** 10.1371/journal.ppat.1014015.r001

**Authors:** Shou-Wei Ding, David Mackey

17 Apr 2026

Two domesticated species of rice shaped the population structure of Xanthomonas oryzae pv. oryzae in Africa

PLOS Pathogens

Dear Dr. Perez-Quintero,

Thank you for submitting your manuscript to PLOS Pathogens. After careful consideration, we feel that it has merit but does not fully meet PLOS Pathogens's publication criteria as it currently stands. The three reviewers agree that this work is a valuable contribution but also make many valid criticisms, several of which point to overinterpretation of the findings. Please address each (with the exception noted below) of the reviewer’s concerns in a modified version, with thorough editing for clarity and consideration to move some of the more speculative parts to the discussion. While they would be welcome additions, the reciprocal inoculation experiments suggested by reviewer #1 and the tests of pathogenicity with TALE variation suggested by reviewer #3 are not a requirement for the revision. We invite you to submit a revised version of the manuscript that addresses these points.

Please submit your revised manuscript by Jun 16 2026 11:59PM. If you will need more time than this to complete your revisions, please reply to this message or contact the journal office at plospathogens@plos.org. When you're ready to submit your revision, log on to https://www.editorialmanager.com/ppathogens/ and select the 'Submissions Needing Revision' folder to locate your manuscript file.

Please include the following items when submitting your revised manuscript:

* A letter that responds to each point raised by the editor and reviewer(s). You should upload this letter as a separate file labeled 'Response to Reviewers'. This file does not need to include responses to any formatting updates and technical items listed in the 'Journal Requirements' section below.

* A marked-up copy of your manuscript that highlights changes made to the original version. You should upload this as a separate file labeled 'Revised Manuscript with Track Changes'.

* An unmarked version of your revised paper without tracked changes. You should upload this as a separate file labeled 'Manuscript'.

If you would like to make changes to your financial disclosure, competing interests statement, or data availability statement, please make these updates within the submission form at the time of resubmission. Guidelines for resubmitting your figure files are available below the reviewer comments at the end of this letter.

We look forward to receiving your revised manuscript.

Kind regards,

David Mackey

Academic Editor

PLOS Pathogens

Shou-Wei Ding

Section Editor

Editor-in-Chief

PLOS Pathogens

Editor-in-Chief

PLOS Pathogens

orcid.org/0000-0002-7699-2064

**Journal Requirements:**

1) Please ensure that the CRediT author contributions listed for every co-author are completed accurately and in full.

At this stage, the following Authors/Authors require contributions: Soungalo Sarra, and Valérie Verdier. Please ensure that the full contributions of each author are acknowledged in the "Add/Edit/Remove Authors" section of our submission form.

The list of CRediT author contributions may be found here: https://journals.plos.org/plospathogens/s/authorship#loc-author-contributions

2) We do not publish any copyright or trademark symbols that usually accompany proprietary names, eg ©,  ®, or TM  (e.g. next to drug or reagent names). Therefore please remove all instances of trademark/copyright symbols throughout the text, including:

- ® on pages: 22, 28, 30, 31, 34, and 36.

3) Please amend your detailed Financial Disclosure statement. This is published with the article. It must therefore be completed in full sentences and contain the exact wording you wish to be published.

- State the initials, alongside each funding source, of each author to receive each grant. For example: "This work was supported by the National Institutes of Health (####### to AM; ###### to CJ) and the National Science Foundation (###### to AM)."

- State what role the funders took in the study. If the funders had no role in your study, please state: "The funders had no role in study design, data collection and analysis, decision to publish, or preparation of the manuscript.".

If you did not receive any funding for this study, please simply state: u201cThe authors received no specific funding for this work.u201d

4) Figures 1 and S1: please (a) provide a direct link to the base layer of the map (i.e., the country or region border shape) and ensure this is also included in the figure legend; and (b) provide a link to the terms of use / license information for the base layer image or shapefile. We cannot publish proprietary or copyrighted maps (e.g. Google Maps, Mapquest) and the terms of use for your map base layer must be compatible with our CC BY 4.0 license.

Note: if you created the map in a software program like R or ArcGIS, please locate and indicate the source of the basemap shapefile onto which data has been plotted.

If your map was obtained from a copyrighted source please amend the figure so that the base map used is from an openly available source. Alternatively, please provide explicit written permission from the copyright holder granting you the right to publish the material under our CC BY 4.0 license.

If you are unsure whether you can use a map or not, please do reach out and we will be able to help you. The following websites are good examples of where you can source open access or public domain maps:

* U.S. Geological Survey (USGS) - All maps are in the public domain. (http://www.usgs.gov)

* PlaniGlobe - All maps are published under a Creative Commons license so please cite “PlaniGlobe, http://www.planiglobe.com, CC BY 2.0” in the image credit after the caption. (http://www.planiglobe.com/?lang=enl)

* Natural Earth - All maps are public domain. (http://www.naturalearthdata.com/about/terms-of-use/)

**Reviewers' Comments:**

Reviewer's Responses to Questions

**Part I - Summary**

Please use this section to discuss strengths/weaknesses of study, novelty/significance, general execution and scholarship.

Reviewer #1: This manuscript offers a significant contribution to understanding Xoo diversity and the evolution of a rice pathogen in Africa. The addition of the whole-genome sequencing AfXoo dataset is a novel contribution. However, the paper suffers from speculative claims about what their data shows. In addition, the focus is currently limited, and could benefit from discussion of literature outside rice pathogens and Xoo to general impacts of allopatric pathogen evolution, or host introductions on pathogen evolution.

The claim about distinct lineages was well supported with NVTR and WGS data. However, there were repeated claims that clonal lineages BAPS 1, 2, and 3 were derived from recombinant lineage 4. Based on the phylogenetic trees presented (esp. Figure 3B), baps 1 is a sister lineage to baps 2, 3, and 4. I do not think the phylogenetic data shows any evidence of the derived population claim for lineage 1 specifically. The diversity in TALE and Xop genes were well presented and documented, but I found claims about recombination in TALE sequences to be unsubstantiated (no actual tests for recombination).

My major issue with this paper was the lack of evidence demonstrating a link between the rice cultivars present in West Africa and pathogen evolution. Throughout the results section, there were speculations about the introduction of O. sativa and pathogen host specialization from native to an introduced rice species. I do not think there is any concrete data supporting this, and these speculations were overstated and presented as a main finding of the manuscript.

Overall, this paper has interesting and novel data, but makes claims beyond what the data shows. In addition, the manuscript suffered from various typos (i.e. ric instead of rice, hos instead of host) and a lack of copyediting throughout.

Reviewer #2: This manuscript by Quibod et al presents a comprehensive and carefully executed population genomics analysis of African Xanthomonas oryzae pv. oryzae (AfXoo), integrating phylogenomics, recombination-aware analyses, demographic inference, and host domestication history. The dataset is extensive, the analytical framework is generally appropriate, and the evolutionary narrative is biologically compelling. My primary concern is overinterpretation of some evolutionary inferences, speculations, and sometimes framing it as causal relationship. Authors can revise their writing and tone down at some places. Overall, the study makes a valuable contribution to understanding pathogen diversification in the context of crop domestication and agroecosystem change.

Reviewer #3: The authors sequence a collection of 59 Xanthomonas oryzae pv. oryzae isolated from Oryza spp. in Africa (AfXoo) to assess their population structure, evolutionary history and diversity of transciption-activator-like effectors (TALEs). A tip-dated tree is shown and demographic inference is performed. TALE identification and repeat domain variation is assessed. Data from pathogenicity assays from previous studies is integrated to draw a link between TALE diversity and rice infection outcomes. Data from a previous study is also integrated to study TALEs diversity in field infections.

**********

**Part II – Major Issues: Key Experiments Required for Acceptance**

Please use this section to detail the key new experiments or modifications of existing experiments that should be absolutely required to validate study conclusions.

Generally, there should be no more than 3 such required experiments or major modifications for a "Major Revision" recommendation. If more than 3 experiments are necessary to validate the study conclusions, then you are encouraged to recommend "Reject".

Reviewer #1: The first issue is speculation about host specialization (AfXoo adaptation to native rice species vs. O. sativa). To empirically back these claims, there needs to be reciprocal inoculation experiments of AfXoo and AsXoo strains in various rice varieties. That would be a massive experiment, but would contribute substantially to the field. In the absence of this (which I doubt is feasible for this manuscript), I think the speculation about host specialization before and after the introduction of O. sativa should be scaled back, removed from all results, and limited to a short paragraph in the discussion. I would suggest a major reframe of the paper, focusing on allopatric pathogen evolution, and to contextualize the results in terms of comparison to the AsXoo population evolution.

The second modification should be an effort to resolve the tree topology of lineages 1, 2, 3, and 4 with improved bootstrap support for critical nodes splitting these lineages. There should be phylogenetic support for a claim that BAPS lineages 1, 2, and 3 are “derived from” 4, or that claim should be removed.

Finally, there should be explicit tests for recombination in TALE and Xop sequences. I would suggest looking at Gubbins, FastGear, or ClonalFrameML to explicitly check for recombination.

Reviewer #2: (No Response)

Reviewer #3: This will be an important resource of general utility and specialist interest, but some changes in data presentation, writing and integration of prior work will strength the quality of the manuscript:

1. The integration of work from previous studies was either patchy (pathogenicity results) or poorly connected to the main results (Diallo et al Bagre field populations). Although both sections ask interesting questions, the prior data is either insufficient (pathogenicity data) or the findings are not sufficiently well placed within the context of the other findings shown here. The Bagre field collection is not described and it is unclear how the results in Fig. 4 are linked with data in Fig. 1-3. This section can be improved with clearer writing and by adding additional detail to relevant figures (e.g. highlighting the 18 Bagre strains in Fig. 1)

2. It is reasonable to hypothesize there is a connection between the arrival of a new Oryza species and the diversification of AfXoo, but I do not see how the coalescent skyline plot and tree provide clear and undisputed evidence of a strong bottleneck in AfXoo corresponding to O. sativa introduction. Some recent clonal expansions were sampled, but a diverse recombinant population is also still circulating. The fact isolates are recovered from other Oryza species - including a wild species - suggests the situation is more complex.

3. Experimental data showing differences in pathogenicity due to variation in TALE alleles would be highly valuable of course.

**********

**Part III – Minor Issues: Editorial and Data Presentation Modifications**

Please use this section for editorial suggestions as well as relatively minor modifications of existing data that would enhance clarity.

Reviewer #1: General notes

The code is not publicly available - the link is not accessible.

The citations were clearly from Zotero, but still linked. Unlink citations from Zotero for submissions, and also the font / size of the citations was different from the surrounding text.

The figure legends dropped in the middle of the text was confusing.

Figures

Figure 1: It says in the legend that it was constructed with 1,000 bootstrap, but there are no labels with bootstrap support for individual nodes. What was the outgroup for this? The white boxes for host (presumably “unknown”?) are not defined. In B, the pie charts are cut off. The country names are included for East Africa but not West Africa

Figure 3: This the phylogenetic tree that makes me suspicious of the claim that lineages 1, 2 and 3 are derived from BAPS 4.

Figure 4A has no x-axis label.

Figure S1 - can’t read text labels for Mali and Niger. Also clarify in the figure legend that the 333 strains were only included for the VNTR testing, while 59 were using in WGS.

Figure S2 - use the same colors for each country that were used in Fig. 1 and Fig. S1.

Figure S3 - There are discrepancies in groupings between DAPC and BAPS that are not addressed anywhere.

Figure S5 - BAPS-5 is included here but not above in S4 (i.e. strain Mai134). Why?

Figure S7 - The ‘highlight’ for strain Mai134 that is mentioned in the figure legend is missing. For C, there are three nodes with dates, but it is not clear why these nodes were selected?

Figure S10 - how was this recombination inferred? The presence of a shared sequences does not necessarily prove recombination.

Results

The logic that lineages 1, 2, and 3 have lower r/m and therefore may be derived from lineage 4 (with higher r/m) does not follow. It is possible that higher r/m in lineage 4 is from introgression from lineages 1, 2, or 3, hence has greater diversity - not that it has greater diversity because it is the ancestral lineage.

Lines 280-289 - only present what your actual data shows. That is, the inferred Xoo population sizes over time. All speculation about the influence of rice species should be moved to the discussion.

Lines 324- 329 - mostly an explanation of background and other studies, should be moved to introduction.

Lines 337 - 338 - no explanation of how recombination was identified.

Lines 370-374 speculation

Lines 396-398 - IR24 and IRBB are not defined - please explain for non-rice specialists.

Lines 403-405, 408-410 - not results, should be moved to introduction or discussion

Discussion

Line 436 - missing parentheses after “ago” i.e. (9,000 years ago)

Line 437 - The evolution of a highly aggressive phenotype is not a predetermined outcome. There is selection for higher fitness, but not always higher aggressiveness.

Line 438 - “In africa” the “In” is capitalized incorrectly

Line 435-445 remove the word “evidence” - this is a suggestion for this hypothesis, not evidence

Line 461-465 - “We can imagine a scenario… “ speculation

Line 482 - “ric” instead of “rice”

Line 521 “While the changes..” this is not a complete sentence.

Line 526 “Here, we demonstrate…” remove “demonstrate” and replace with “hypothesize”

Line 528 - “adapted” - was not actually shown

Methods

There were two different methods using for core genome SNP extraction. The first (for the entire Xo species) was using panaroo and snp-sites, and then after for Afxoo, the method changed to using parsnip and gubbins. This is not justified, and it is confusing to use two distinct methods

Line 611 - which SNP alignment was used for Splits tree?

In general, clarify when the core genome SNP matrix was used with recombination or without recombination

Line 640 - 660 - the inclusion of Mai134 or not is confusing throughout this. There are either four or five k / populations, but it is unclear why Mai134 was included or not in each of these analyses.

Line 658 - Fst - pairwise?

Lines 674-677 - missing details on which rice genes?

Reviewer #2: Below are my comments/suggestions.

Abstract: line 82-84. Is it evolutionary force acting upon rice? Or other way around?

Fig S1 could benefit from having years of isolation indicated alongside country and number of strains. It is great that authors have used different figures to explain the vast dataset, but sometimes, looking at the map and knowing the timespan in which strain collection is done is helpful.

Fig 3a: Yellow points- are those AfXoo strains clustering with Asian Xoo? I assume they are not in subsequent analyses? If included, would there be shared alleles among those strains and any AfXoo lineages?

My primary concerns are overinterpretation of some evolutionary inferences, speculations, and sometimes framing it as causal relationship. Reframing these or perhaps, stating the alternative explanations would be beneficial for the readers. Below are some of these examples:

1. Line 246-247. How is this evolutionary trajectory inferred about adapting different host resistances? Is this based on phenotypic data?

2. Inferred ages on the tip-dating analysis primarily reflect allele histories rather than lineage emergence per se.

3. BAPS4 is polyphyletic, and shares alleles with other BAPS. This lineage is ancestral-like recombining reservoir from which multiple monophyletic, clonal lineages emerged and this is supported by the data presented by the authors. This is a reasonable and supported explanation. However, authors need to be cautious about using a term founder population. Observation that BAPS4 strains exhibit no virulence in any of the tested lines, despite persisting in the populations and being largely associated with O. sativa (only 1 strain from other Oryza)- this is intriguing and warrants a brief discussion. Even if ancestral-like, the long-term persistence of these avirulent (?) strains can mean some interesting ecological fitness factors? Were these strains pathogenic on the host of isolation?

4. Another statement: introduction of O. sativa into Africa served as a bottleneck in AfXoo population is another place where narrative is somewhat appearing as causal. While demographic contractions inferred are consistent with domestication of two rice species, coalescent based effective population size estimates alone cannot establish the causation. Alternative explanations such as sampling bias, or changes in agricultural practices other than host, or climate or combination thereof, remain plausible. So more cautious formulation might be useful here to show biological interpretation by the authors.

5. TalF variants in BAPS1 lacking virulence. Is this demonstrated only in one strain or multiple? How are authors coming to the inference that TalF has no role in virulence?

6. Mali as center for diversification: Isn’t Mali heavily sampled here? How can it be distinguished from true evolutionary origin?

Reviewer #3: Clarity of text from L117 onwards can be improved, there are abundant sentence fragments and strange sentence constructions. Some examples:

L118 - sentence begins with 'And'

L128 - 'TALE diversification ... has been shown to play an active role in shaping the diversity of TALEs'. Circular.

L172 - 'These strains formed a single node'. Meaning they are monophyletic?

L334 - 'The RVD alignment also showed indels in some TALEs, and could go as long as seven to two gaps'. Improve phrasing.

L396 - 'BAPS3 and 4, were unable to infect IR24, an AsXoo susceptibility check, as this particular rice background was shown to contain the Xa18 resistance gene'. Improve phrasing.

L432 - 'fastly spreading'

L438 - 'Meanwhile, In Africa, domestication of O. glaberrima was relatively recent (3,000 years ago), and this is likely reflected in its genetic diversity, with O. glaberrima having less genetic variation compared to O. sativa'. Improve phrasing.

L628 - 'The detailed parameters for TreeTime are resolving polytomies, calculation of confidence intervals by marginal posterior distribution, and maximal iteration of 1000 for maximum optimization.' Those are not detailed parameters.

***

175 - BAPS4 is very interesting. The cluster includes recombinant genomes and is polyphyletic in the tree. The differences for BAPS4 in diversity and Tajima's D are unsurprising given these are recombinant genomes.

L185 - No support values are shown on the tree in Fig. 1. Some unresolved relationships and lack of support evident in Fig. S3. Figure display for S3 can be improved.

L197 what is meant by BAPS2 emergence coinciding with loss of BAPS3? Were BAPS2 and BAPS3 isolated from the same precise location in Mali? BAPS2 looks like a recent clonal expansion in Fig.1, if these were all sampled from one location in Mali it is likely BAPS2 recently spread in that area.

L229 - Recent expansion is very likely given Fig. 1, and recombination is more likely than balancing selection given the data shown. Strange dashed scale bars.

L239 - BAPS1-3 have recovered more rapidly from what?

L246 - It is speculative to conclude the presence of unique alleles in a population is due to their adaptation to different host genetic resistances or niches, this could be drift.

L264 - 'reconstruction revealed that the emergence of the AfXoo group occurred around the year 656 CE (common era) (334 BCE-1351 CE 95%HPD) during the period of intensive cultivation of African rice O. glaberrima'. The period referred to here is more appropriately the entire 1000 year interval predating O. sativa introduction.

L280 - Rather than a recent bottleneck on O. sativa, is it possible the apparent reduction is due to having sampled 3 recently expanded clonal lineages that share distant ancestry with each other? Coalescent inference of past demography (along with tip dating) produces statistical ranges based on underlying assumptions about the nature of the data.

L311 - 'a single BAPS4 isolate that contains exactly the same TALE family allele with some BAPS1 strains' - this is interesting, worth pursuing experimentally.

L314 / Fig. 4a - Are there alternative methods of visually summarising TALE diversity? An alternative to a colored table must be possible

L323 - whole paragraph of speculation about the absence of iTALES in the African Xfoo pathogen population and their presence in a different Asian population, which is not the subject of this paper. Move to Discussion.

L343 - Here the paper takes a strange turn. Authors explain they examined the spatiotemporal resolution of TALE effectors in the field, by 'reexamining data' from another paper. It is not clear what connection the data in that paper has to the work shown up to this point. Where is Bagre relative to the sample areas in Fig. 1?

L349 - New strains were sequenced from Bagre?

L355 - Were all 18 Bagre strains already shown in Fig. 1? Which are these exactly among the 30 Burkina Faso genomes? Membership in BAPS cluster 1 is mentioned but the relatedness or identity between the 18 Bagre isolates from Diallo et al. to the 59 sequenced for Fig. 1-4 is not clearly explained. I understand the idea in principle, but often struggled to connect the data from Diallo et al. / Fig. 4 to Fig. 1-3.

Which of the (e.g.) six TalA alleles in Fig. 4a correspond to the two TalA alleles in Fig. 5a? How is the TALome pattern in Fig. 5a linked with the TALE alleles in Fig. 5a? Why does CIX3092 have TALome pattern 2 and not 5?

TALE allele presence for 8 genomes is shown in Fig. 5a. Since these strains appeared the tree in Fig. 1, is the data for the 10 additional strains shown only in the Fig. 5b barchart? There are 30 Burkina Faso genomes in Fig. 1 - are the 18 Bagre isolates a subset? A partially different set? Statements like 'Since the strains analyzed share the same node (S3b Fig),' are difficult to interpret: presumably this means the BAPS1 isolates are monophyletic, but which node? There are several, 'same node' could mean very different things across the tree.

L393 - The last section also compiled some pathogenicity assay results from multiple studies to draw conclusions about how TALome influences infection in the field. This is more easily integrated this paper but given the heterogeneous sources of this data and patchy TALome representation, I think it is mainly useful for hypothesis generation.

L398 - What are IRBB lines? What are xa5 and xa7?

Methods L547 - the summary of integration of pathogenicity assay from other studies should be in a different Methods section.

**********

PLOS authors have the option to publish the peer review history of their article (what does this mean?). If published, this will include your full peer review and any attached files.

If you choose “no”, your identity will remain anonymous but your review may still be made public.

**Do you want your identity to be public for this peer review?**  For information about this choice, including consent withdrawal, please see our Privacy Policy.

Reviewer #1: No

Reviewer #2: No

Reviewer #3: No

[NOTE: If reviewer comments were submitted as an attachment file, they will be attached to this email and accessible via the submission site. Please log into your account, locate the manuscript record, and check for the action link "View Attachments". If this link does not appear, there are no attachment files.]

**Figure resubmission:**

**Reproducibility:**

?>

# Author comment: Author response to Decision Letter 1

**DOI:** 10.1371/journal.ppat.1014015.r002

17 Aug 2026

Attachment Submitted filename: Response_to_reviewers.docx

# Editor report: Decision Letter 1

**DOI:** 10.1371/journal.ppat.1014015.r003

**Authors:** Shou-Wei Ding, David Mackey

27 Aug 2026

Dear Mr Perez-Quintero,

We are pleased to inform that your manuscript, "Two domesticated species of rice shaped the population structure of Xanthomonas oryzae pv. oryzae in Africa", has been editorially accepted for publication at PLOS Pathogens.

Your revisions addressed nearly all of the reviewers concerns and, in the one case where you didn't, I concur that the patho-testing is beyond the scope of this manuscript. Some clunky language use/grammar persists, so I urge you to work closely with copy editor during the final revisions. Congratulations!

Before your manuscript can be formally accepted and sent to production, you will need to complete our formatting changes, which you will receive by email within a week. Please note that your manuscript will not be scheduled for publication until you have made the required changes.

IMPORTANT NOTES

(1) Copyediting and Proofreading: The corresponding author will receive a typeset proof for review, to ensure errors have not been introduced during production. Please review the PDF proof of your manuscript carefully, as this is the last chance to correct any errors. Please note that major changes, or those which affect the scientific understanding of the work, will likely cause delays to the publication date of your manuscript.

(2) Appropriate Figure Files: Please remove all name and figure # text from your figure files. Please also take this time to check that your figures are of high resolution, which will improve the readbility of your figures and help expedite your manuscript's publication. Please note that figures must have been originally created at 300dpi or higher. Do not manually increase the resolution of your files. For instructions on how to properly obtain high quality images, please review our Figure Guidelines, with examples at: http://journals.plos.org/plospathogens/s/figures.

We encourage you to use PACE (http://pace.apexcovantage.com/), a figure-checking tool which helps authors ensure all figures meet PLOS requirements so that figures can publish in the highest quality. PACE is a digital diagnostic and conversion tool for figure files. It will provide information about any failed check(s) and, if able, will automatically convert the figure into an acceptable file that passes quality checks.

To use PACE, you must first register as a user. Then, login and navigate to the UPLOAD tab, where you will find detailed instructions on how to use the tool.

(3) Striking Image: Please upload a striking still image to accompany your article if one is available (you can include a new image or an existing one from within your manuscript). Should your paper be accepted, this image will be considered for our monthly issue image and may also appear on our website to feature your article. Please upload this as a separate file, selecting "striking image" as the file type upon upload. Please also include a separate "Other" file with a caption, including credits and any potential copyright information. Please do not include the caption in the main article file. If your image is from someone other than yourself, please ensure that the artist has read and agreed to the terms and conditions of the Creative Commons Attribution License at http://journals.plos.org/plospathogens/s/content-license. Please note that PLOS cannot publish copyrighted images.

(4) Press Release or Related Media: If you or your institution will be preparing press materials for this manuscript, please inform our press team in advance at plospathogens@plos.org as soon as possible. We ask that you contact us within one week to plan ahead of our fast Production schedule. If you need to know your paper's publication date for related media purposes, you must coordinate with our press team, and your manuscript will remain under a strict press embargo until the publication date and time.

(5) Update your Profile Information: Now that your manuscript has been provisionally accepted, please log into Editorial Manager and update your profile, if needed. Go to http://www.editorialmanager.com/ppathogens, log in, and click on the "Update My Information" link at the top of the page. Please update your user information to ensure an efficient production and billing process.

(6) LaTeX users only: Our staff will ask you to upload a TEX file in addition to the PDF before the paper can be sent to typesetting, so please carefully review our Latex Guidelines http://journals.plos.org/plospathogens/s/latex in the meantime.

Thank you again for supporting Open Access publishing; we are looking forward to publishing your work in PLOS Pathogens.

Best regards,

David Mackey

Academic Editor

PLOS Pathogens

Shou-Wei Ding

Section Editor

PLOS Pathogens

Dominique Soldati-Favre

Editor-in-Chief

PLOS Pathogens

***********************************************************

# Editor report: Acceptance letter

**DOI:** 10.1371/journal.ppat.1014015.r004

**Authors:** Shou-Wei Ding, David Mackey

Dear Mr Perez-Quintero,

We are delighted to inform you that your manuscript, "Two domesticated species of rice shaped the population structure of Xanthomonas oryzae pv. oryzae in Africa," has been formally accepted for publication in PLOS Pathogens.

We have now passed your article onto the PLOS Production Department who will complete the rest of the pre-publication process. All authors will receive a confirmation email upon publication.

Thank you again for supporting open-access publishing; we are looking forward to publishing your work in PLOS Pathogens.

Best regards,

Dominique Soldati-Favre

Editor-in-Chief

PLOS Pathogens
